# Chemotherapy induces dynamic immune responses in breast cancers that impact treatment outcome

**DOI:** 10.1038/s41467-020-19933-0

**Published:** 2020-12-02

**Authors:** Yeon Hee Park, Samir Lal, Jeong Eon Lee, Yoon-La Choi, Ji Wen, Sripad Ram, Ying Ding, Soo-Hyeon Lee, Eric Powell, Se Kyung Lee, Jong Han Yu, Keith A. Ching, Jae-Yong Nam, Seok Won Kim, Seok Jin Nam, Ji-Yeon Kim, Soo Youn Cho, Seri Park, Jinho Kim, Soohyn Hwang, Yu Jin Kim, Vinicius Bonato, Diane Fernandez, Shibing Deng, Shuoguo Wang, Hyuntae Shin, Eun-Suk Kang, Woong-Yang Park, Paul A. Rejto, Jadwiga Bienkowska, Zhengyan Kan

**Affiliations:** 1 Samsung Medical Center, Seoul, Korea; 2 Oncology Research & Development, Pfizer, San Diego, CA USA; 3 Drug Safety R&D, Pfizer, San Diego, CA USA; 4 Pfizer Oncology, Seoul, Korea; 5 Samsung Genome Institute, Samsung Medical Center, Seoul, Korea; 6 Biostatistics, Pfizer, San Diego, CA USA

**Keywords:** Breast cancer, Cancer genomics, Chemotherapy

## Abstract

To elucidate the effects of neoadjuvant chemotherapy (NAC), we conduct whole transcriptome profiling coupled with histopathology analyses of a longitudinal breast cancer cohort of 146 patients including 110 pairs of serial tumor biopsies collected before treatment, after the first cycle of treatment and at the time of surgery. Here, we show that cytotoxic chemotherapies induce dynamic changes in the tumor immune microenvironment that vary by subtype and pathologic response. Just one cycle of treatment induces an immune stimulatory microenvironment harboring more tumor infiltrating lymphocytes (TILs) and up-regulation of inflammatory signatures predictive of response to anti-PD1 therapies while residual tumors are immune suppressed at end-of-treatment compared to the baseline. Increases in TILs and CD8+ T cell proportions in response to NAC are independently associated with pathologic complete response. Further, on-treatment immune response is more predictive of treatment outcome than immune features in paired baseline samples although these are strongly correlated.

## Abstract (web summary)

Neoadjuvant chemotherapy is a therapeutic option for the treatment of breast cancer. Here, the authors characterize changes in the gene expression profiles and immune microenvironment in serial breast cancer biopsies taken before, during and after neoadjuvant chemotherapy.

## Introduction

Despite tremendous advances in targeted and immuno-oncology (IO) therapies, cytotoxic chemotherapies remain the backbone of treatment for many cancers at different stages of the disease^1^. Chemotherapy has long been regarded as immune suppressive due to dose-limiting myelosuppression, however, there is mounting evidence that the efficacy of chemotherapies does not only involve cell intrinsic cytotoxic effects but also relies on activating antitumor immune responses^2^. Efforts are underway to combine chemotherapies with IO therapies to harness the synergistic effects based on the hypothesis that chemotherapies provide a broad-acting immune stimulus against cancers^3–5^. The design of novel therapy as well as optimal combination strategy require detailed understanding of the effects of chemotherapies on tumor cell intrinsic biology as well as tumor microenvironment. To date, the tumor associated immunomodulatory effects and mechanisms of chemotherapeutic treatments are mainly studied in mouse models^2^. There is a lack of comprehensive and systematic characterization of the effects of chemotherapy on the tumor immune milieu in the clinical setting, especially on-treatment or post-treatment. Preclinical observations of drug treatment responses may be discordant with clinical findings. One study reported immune stimulatory effects of cyclophosphamide treatment in tumor bearing mice by performing gene and protein expression profiling, and proposed the optimal timing for combination with IO therapy to be approximately one day after the initial chemotherapy treatment^6^. However, serial expression analyses of breast tumors from patients receiving neoadjuvant chemotherapies (NAC) in the I-SPY 1 trial presented contradictory evidence, revealing downregulation of immune genes after 1–4 days of chemotherapy treatment^7^.

The challenge of obtaining post-treatment clinical samples is the main reason for the dearth of molecular profiling data available for studying tumor responses to standard-of-care treatments such as chemotherapy^8,9^. Neoadjuvant treatment where patients receive systematic therapy before surgical removal of the tumor is an attractive setting for assessing drug effects on targets, tumor biology, and identifying molecular markers of clinical outcome^10–12^. In breast cancer (BC), particularly the ER-negative subtypes, neoadjuvant chemotherapy is the preferred treatment approach for locally advanced cancers with pathologic complete response (pCR) being the primary endpoint associated with improved prognosis^13–16^. The combination of anthracyclines and taxanes with the addition of trastuzumab for the HER2+ subtype has been the backbone regimen in BC in the adjuvant and neoadjuvant setting for years^17–19^. Despite successes of IO therapies in the treatment of many cancers, including the metastatic TNBC^20^, identifying beneficial treatment regimens for BC patients that take advantage of IO therapies remains a challenge. Checkpoint inhibitors targeting the programmed cell death protein 1 (PD1) and PD1 ligand 1 (PDL1) are clinically validated^21^ and form the foundation for IO combination strategies, where the design is guided by markers such as PDL1 expression and immune pathway specific mRNA profiles^22^. Histological analyses of pre-treatment and post-treatment tumor specimens from 25 BC patients have revealed correlation between the development of tumor infiltrating lymphocytes (TILs) after neoadjuvant paclitaxel chemotherapy and clinical response^23^. Several studies have reported clinical implications for baseline TIL counts and PDL1 positivity after evaluation of pre-treatment and post-treatment samples in the NAC settings using immunohistochemistry and gene expression profiling^24–26^. However, these studies mainly focused on tumor biopsies taken before and after the NAC treatment, but did not sufficiently characterize the changes that occur in tumor tissues during treatment. A recent expression profiling study of 97 sequential samples from a cohort of 50 breast cancer patients, who had been treated by NAC reported that on-treatment biomarkers can improve prediction of NAC response but did not identify any changes in immune gene expression^27^.

We hypothesized that comprehensive profiling of serial tumor biopsies taken before, during and after neoadjuvant treatment can delineate the impact of NAC on the immune microenvironment and may guide the selection of IO therapies and rational design of combination^22^. In this study, we perform whole transcriptome profiling coupled with histopathology analyses of serial samples at three time points from 146 breast cancer patients undergoing neoadjuvant chemotherapy treatment. We then perform systematic analyses to identify NAC induced changes in gene expression profiles and immune microenvironment, as well as examined associations between molecular attributes and clinical outcome. Here, we report that the first cycle of neoadjuvant chemotherapy induces an immune stimulatory response in tumors, that is independently associated with pathologic complete response and more predictive of treatment outcome than baseline immune features. Our findings suggest that the effectiveness of neoadjuvant chemotherapies may be improved by combining with immunomodulatory therapies in the early stage of the treatment while assessing tumor-associated immune responses during treatment can help identify patients likely to benefit from the IO combination.

## Results

### Overview of study design and data

**Fig. 1:**
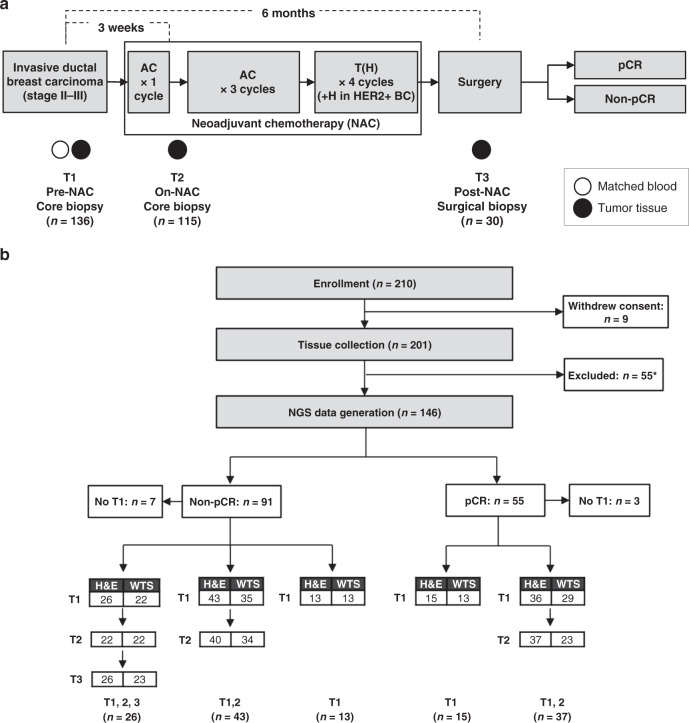
Study design and consort diagram. **a** Study design diagram. AC Adriamycin (Doxorubicin) + Cyclophosphamide, T Taxol (Docetaxel), H Herceptin (Trastuzumab), NAC neoadjuvant chemotherapy, pCR pathologic complete response. (**b**) Consort diagram for patient enrollment, sample collection and data generation. Tissues were not collected for nine patients who withdrew consent. *55 patients were excluded from NGS data generation due to tissue collection failure (33), follow-up failure (7), withdrawal of consent (6), non-breast cancer pathology (4), distant progression and exclusion from surgery (3), NAC termination (1) and expiration (1). Two non-pCR patients had paired T1, T3 samples. WTS whole transcriptome sequencing.

Over a period of 2.5 years, we recruited 210 patients diagnosed with invasive breast carcinoma who were treated with standard neoadjuvant chemotherapy (NAC) protocol of 4 cycles of Anthracycline and Cyclophosphamide followed by four cycles of Docetaxel (T) or Docetaxel plus Trastuzumab for HER2+ diseases. Tumor biopsies were collected for each patient at three time points—pre-treatment (T1), three weeks after the first cycle of NAC (T2) and at the time of surgery (T3) for patients who did not achieve pCR (Fig. 1a). We successfully conducted whole transcriptome sequencing (WTS) and histopathology analysis on 281 tumor samples from 146 cases including 110 longitudinal pairs (Fig. 1 and Supplementary Data 1). Of these patients, 55 (38%) achieved pCR while 91 (62%) harbored residual disease (RD). RNA-Seq was performed on 227 tumor samples from 136 patients, consisting of 17 triplets (T1–T2–T3) and 57 pairs (T1–T2, T1–T3, or T2–T3) with coverage for all paired samples (Fig. 1b). T1 baseline samples were profiled for 112 patients consisting of 42 pCR cases (37.5%) and 70 RD cases (62.5%). Paired T1 and T2 samples were profiled for a subset of 68 patients consisting of 21 pCR cases and 47 RD cases. Hematoxylin and eosin (H&E) stained slides were available for 271 tumor samples from 146 patients. We classified all RNA-Seq profiled samples into four subtypes—ER+ /HER2−, ER+ /HER2+, HER2+ /ER− and triple negative (TN) mainly based on immunohistochemistry (IHC) analyses of ER, PR, and HER2 markers. According to subtype classification of pre-treatment samples, 22% (30/136) of the cohort were ER+ /HER2−, 20% (27/136) were ER+ /HER2+, 20% (27/136) were HER2+ /ER− and 38% (52/136) were TN (Supplementary Table 1).

### Differential regulation of cancer hallmark pathways during NAC

**Fig. 2:**
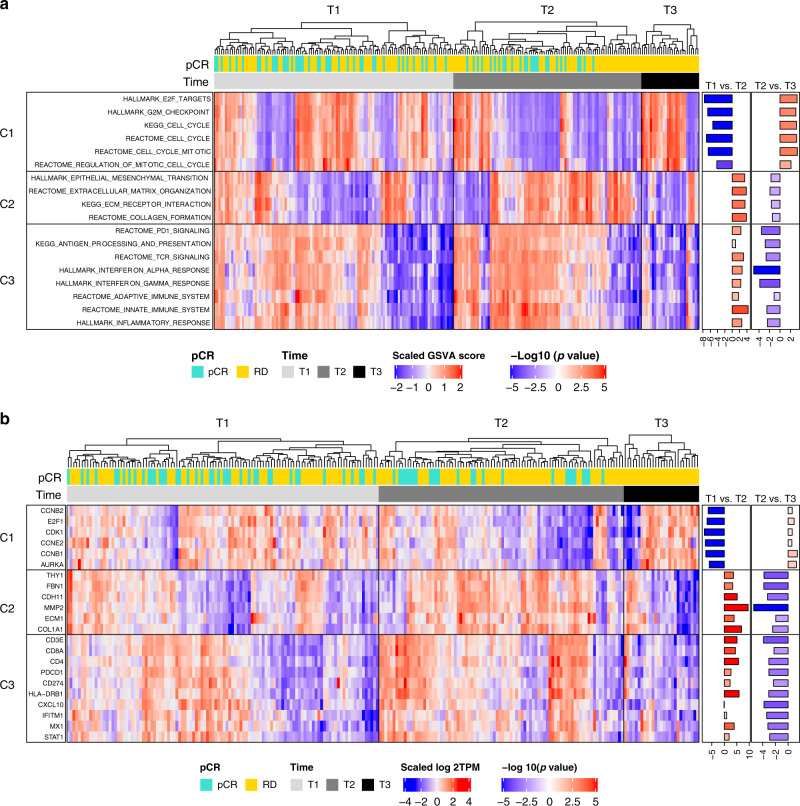
Differential expression patterns across treatment times. Expression patterns of representative pathways (**a**) and genes (**b**) mapped to the three DE gene clusters. GSVA scores in **a** were centered and scaled to *z*-scores for each pathway geneset. Statistical significance of DE was determined using linear mixed-effects regression analysis (LMER) and shown as sign*−log10(*p*-value) based on the sign of the *t*-statistics. Source data are provided as a Source Data file.

Global differential expression (DE) analysis identified three consensus clusters of 1987 DE genes that form contrasting DE patterns over treatment times and enriched in cell cycle (C1), epithelial mesenchymal transition (EMT), and extracellular matrix (ECM) (C2) and immune (C3) pathways that are major cancer hallmarks (Supplementary Fig. 1a and Supplementary Data 2). Using the GSVA algorithm^28^ to calculate gene expression signature scores, we confirmed that pathways significantly enriched in the DE gene and clusters as well as representative genes in these pathways also exhibited consistent expression patterns over time (Fig. 2a, b and Supplementary Data 3). We further examined the differential expression patterns of key genes and pathways mapped to the DE clusters in different breast cancer subtypes (Supplementary Figs. 1, 2). In all subtypes, the first cycle of NAC treatment induced downregulation of cell-cycle genes exemplified by pathways related to cell growth and proliferation, such as the Hallmark E2F targets signature. However, cell cycle related gene expressions rebounded to higher levels in residual tumors at surgery time, with the most striking increases observed in HER2+ and TNBC (Fig. 2). On the other hand, the first cycle of NAC induced upregulation of immune and EMT/ECM-related pathways in all subtypes. These pathway expressions then decreased to below baseline levels at end of treatment in HER2+ and TN subtypes (Fig. 2). These dynamic patterns of upregulation and downregulation seemed to be driven by changes in tumor cellularity reflecting the clinical observation, that chemotherapies initially elicit tumor shrinkage yet cancers remain refractory in most patients following treatment.

**Fig. 3:**
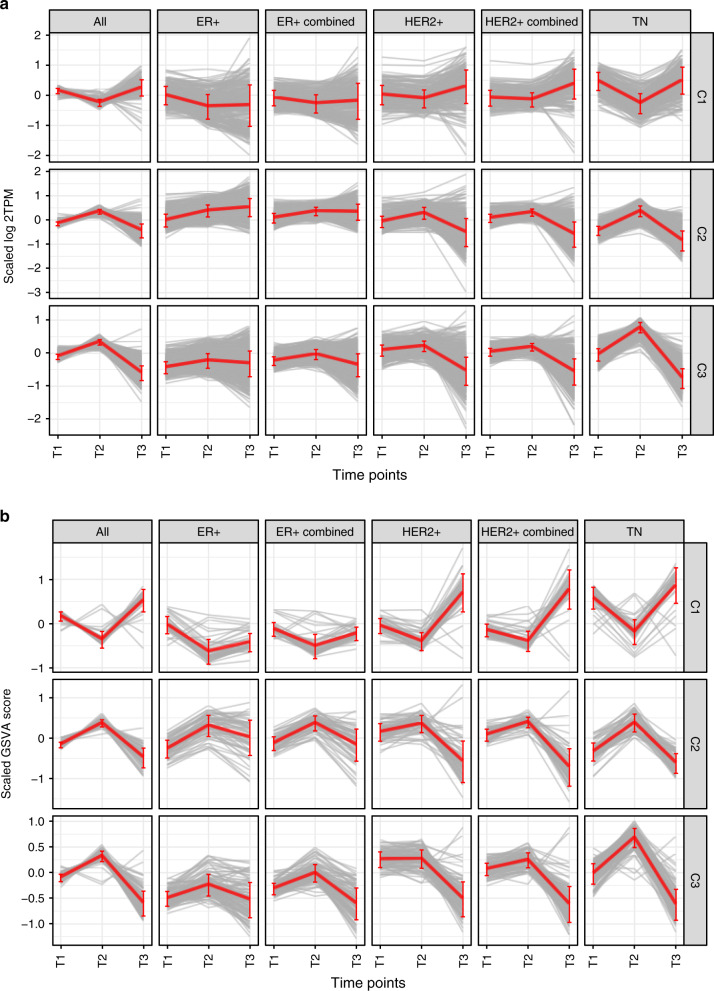
Comparing differential expression patterns across subtypes. **a** Aggregate expression patterns of three DE gene clusters in the overall cohort and subtypes. Gray lines represent individual gene expressions and red lines represent the summarized scores (GSVA) for each cluster. The ER+/HER2+ subtype was excluded due to a lack of T3 samples. “ER+ combined” includes ER+ and ER+/HER2+ subtypes. “HER2+ combined” includes ER+/HER2+ and HER2+ subtypes. Error bars represent the standard deviation of the scaled log2TPM value at each time point. Sample sizes used in the analysis are the following. All: *n* = 112 (T1), *n* = 88 (T2), *n* = 27 (T3); ER+: *n* = 26 (T1), *n* = 18 (T2), *n* = 7 (T3); ER+ Combined: *n* = 49 (T1), *n* = 36 (T2), *n* = 8 (T3); HER2+: *n* = 19 (T1), *n* = 17 (T2), *n* = 5 (T3); HER2+ Combined: *n* = 42 (T1), *n* = 35 (T2), *n* = 6 (T3); TN: *n* = 44 (T1), *n* = 35 (T2), *n* = 14 (T3). Source data are provided as a Source Data file. **b** Aggregate expression patterns of pathways mapped to the three DE clusters in the overall cohort and subtypes. Gray lines represent individual gene signatures (GSVA scores) and red lines represent the averages for each sample group. Error bars represent the standard deviation of the scaled GSVA score at each time point. Sample sizes used to derive statistics are the same as in Fig. 2a. Source data are provided as a Source Data file.

Differential expression patterns enriched in the three cancer hallmarks appeared to be consistent across subtypes but were more clearly exhibited in the HER2+ and TN subtypes than the ER+ subtype (Fig. 3). We performed an in-depth comparison of DE patterns between different breast cancer subtypes based on the correlations of the statistical significance of the DE events and observed concordant trends at both gene and pathway level (Supplementary Fig. 3). Early DE events (T1 vs. T2) were strongly correlated at the pathway level among subtypes with Spearman correlations of 0.633, 0.744, and 0.633 when comparing ER+ vs. HER2+, ER+ vs. TN, and HER2+ vs. TN, respectively (Supplementary Fig. 3a). In particular, DE patterns of cell cycle related pathways were more strongly correlated than EMT/ECM and immune pathways. On the other hand, late DE pathways (T2 vs. T3) were weakly correlated among subtypes, exhibiting Spearman correlations of 0.114, 0.080, and 0.404 for comparisons of ER+ vs. HER2+, ER+ vs. TN and HER2+ vs. TN (Supplementary Fig. 3b). During this stage, the DE patterns in the ER+ subtype diverged from those in the HER2+ and TN subtypes. For instance, DE events in the ER+ subtype were negatively correlated with those in TN at both gene (rho = −0.09) and pathway level (rho = −0.047) (Supplementary Fig. 3b, e). Notably, DE events from the baseline vs. residual comparison (T1 vs. T3) were anti-correlated between ER+ tumors and HER2+ and TN tumors (ER+ vs. HER2+ : rho = −0.479, ER+ vs. TN: rho = −0.179) (Supplementary Fig. 3c, f). In fact, cell cycle related pathways were down regulated in residual vs. baseline tumors in the ER+ subtype but up regulated in the HER2+ and TN subtypes, indicating that NAC exerted variable effects on growth and proliferation in different subtypes. Hence, chemotherapy-induced molecular responses were largely similar during early stages of treatment but became more distinctive among different breast cancer subtypes at end-of-treatment.

### NAC induced dynamic changes in tumor infiltrating lymphocytes (TILs)

**Fig. 4:**
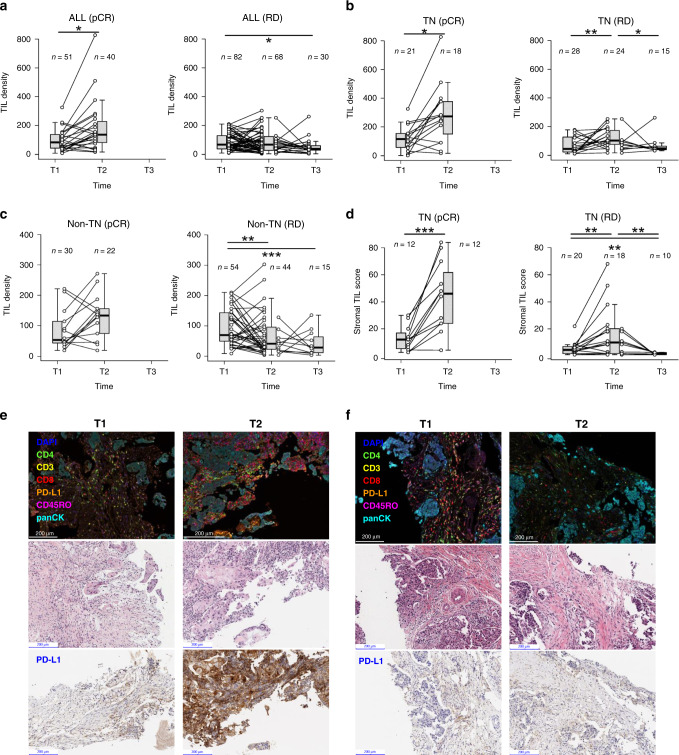
Tumor infiltrating lymphocyte density increased during treatment then decreased below baseline at surgery time. TIL density distributions over three time points were compared between tumors from pCR and RD patients in the overall cohort (**a**), TN (**b**), and non-TN subtypes (**c**). **d** Distribution of stromal TIL scores over time in TN tumors stratified into pCR and RD groups. Asterisks indicate statistical significance based on linear mixed effects regression (LMER) adjusting for tumor purity and subtype as covariates. *0.01 < *p* < 0.05; **0.001 < *p* < 0.01; ***p < 0.001. See Supplementary Table 2 for exact *p*-values. For all box-and-whisker plots, the box is bounded by the first and third quartile with a horizontal line at the median and whiskers extend to the maximum and minimum value. Source data are provided as a Source Data file. **e**, **f** Multiplex immunofluorescence (IF), H&E and PD-L1 IHC images showing the same regions of tumors taken at T1 and T2 from a TNBC patient—BR294 (**e**) and a HER2+ patient—BR308 (**f**). Markers include CD45RO, CD3, CD4, CD8, PD-L1, and Pan-CK. Chromogenic IHC and multiplex IF assays were only performed once on tumor biopsy samples following assay optimization. Scale bars in the lower-left corner of the micrographs show 200 μm.

The dynamic changes in the immune gene expressions suggested that NAC treatment exerted a major impact on tumor infiltrating lymphocytes (TILs), which play an important role in mediating the efficacy of immunomodulatory therapies. To further investigate NAC induced effect on tumor-associated immunity, we quantified the density of TILs by performing digital image analyses of H&E slides and analyzed changes over time (Fig. 4 and Supplementary Data 4, Supplementary Table 2). The majority of tumors exhibited an increase in TIL density at three weeks after the first cycle of NAC followed by a drop to below baseline levels at surgery, with the pattern of change varying across subtypes and pCR status (Fig. 4a). TIL density increases from T1 to T2 were observed in the pCR cohorts across all subtypes, including 80% of cases (12/15) in TN and 64% of cases (14/21) in non-TN subtypes (Fig. 4b, c and Supplementary Table 3). However, among patients with residual disease, TIL densities increased on-treatment in 85% (15/18) of TN cases but decreased in 62% (27/44) of non-TN cases. Pathologists visually inspected the H&E images of TN cases to derive a stromal TIL score based on international standard^29^, and corroborated the up–down pattern of TIL changes over three time points observed in TN (Fig. 4d and Supplementary Fig. 4a). Multiplex IF analysis using a panel of TIL markers on a subset of cases also demonstrated that T cell populations expanded in TNBC upon initial NAC treatment while contracting among non-TNBC cases (Fig. 4e, f).

Gene expression signatures have been reported to predict response to anti-PD1 inhibitor, including a T cell-associated inflammatory signature and an expanded immune signature consisting of individual components that encompass multiple immunomodulatory functions^30^. TN patients with residual disease have significantly shorter overall and post-recurrence survival than non-TN patients^16,31^. We found that NAC induced remarkable on-treatment increases in these IO predictive signatures among TN patients with residual diseases, pointing to a promising approach of combining NAC and checkpoint inhibitors for addressing this unmet medical need (Supplementary Fig. 4b, c).

### Profound impact of NAC on the immune landscape

To gain insights into the NAC induced effects on different TIL subpopulations, we performed a deconvolution analysis of bulk-tumor gene expression^32^ to infer the relative fractions of ten immune cell types among tumor infiltrating leukocytes in each tumor. Strong positive correlations vs. CYT score, a measure of cellular cytolytic activities defined as the geometric mean of *GZMA* and *PRF1* expressions^33^, revealed immune stimulatory roles for CD8+ T, CD4+ memory T cells, and M1 macrophages. Conversely, negative correlations vs. CYT scores indicated that M2 macrophages and mast cells were immune suppressive (Supplementary Fig. 5a). There were significant increases in the relative fractions of CD4+ memory T cells (*p* = 3.18e−06) and CD8+ T cells (*p* = 0.0072) after the first cycle of NAC (Supplementary Fig. 5b and Supplementary Table 4) followed by a decrease in the fractions of CD4+ memory T cells and M1 macrophages between on-treatment and surgery. On the other hand, cell fractions of mast cells and M2 macrophages increased in residual tumors compared to on-treatment samples (Supplementary Fig. 5c). Hence, the first cycle of NAC treatment appeared to induce a stimulatory immune microenvironment in the tumors while the residual tumors harbored a more immune suppressed microenvironment compared to the baseline tumors.

**Fig. 5:**
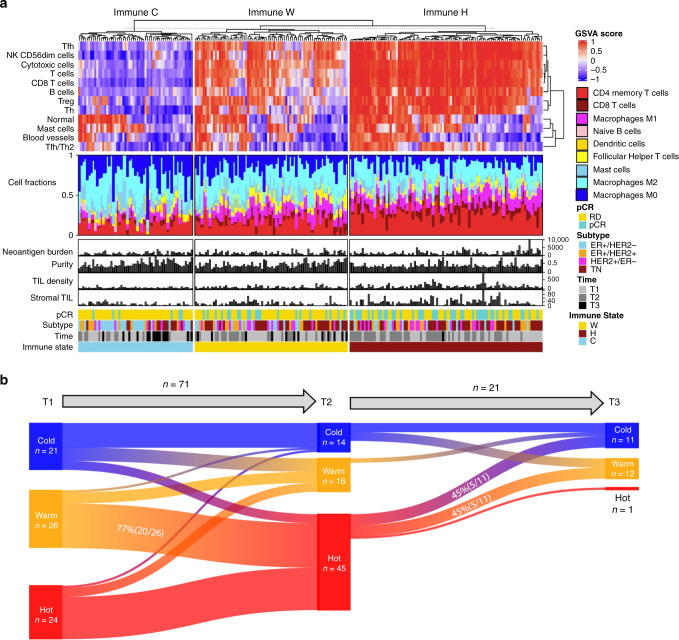
Neoadjuvant chemotherapy induced dynamic changes in tumor associated immune states. **a** Integrated clustering of immune signatures and most abundant immune cell fractions. **b** Sankey plot of immune state changes from T1 to T2 and from T2 to T3. The text denotes the number of samples in each immune state and the percentage of samples that switch from one immune state to another. Source data are provided as a Source Data file.

Unsupervised integrative clustering of immune expression signatures and immune cell fractions clearly classified all samples into three distinct immune states: cold (C), warm (W), and hot (H) (Fig. 5a and Supplementary Data 4). The H state marks an immune stimulated microenvironment harboring higher fractions of CD4+, CD8+ T cells, and M1 macrophages as well as elevated TIL abundance compared to the two other states. The C state marks an immune suppressed microenvironment with higher fractions of M0, M2 macrophages and mast cells than other immune states while the W state appears to be an intermediate between the H and C states (Supplementary Fig. 6a, b). The immune states are significantly associated with subtype (*p* = 1.7e−03), treatment times (*p* = 9.4e−06) and treatment response (*p* = 8.5e−04) (Supplementary Fig. 6c–e and Supplementary Table 5). We observed frequent changes in tumor associated immune states during the course of treatment with 77% of baseline tumors (20/26) classified as W turning into H at T2 and 91% of H tumors (10/11) at T2 switching to W or C at T3 (Fig. 5b). The patterns of immune state transition are significantly associated with breast cancer subtype (*p* = 7.98e−4) (Supplementary Table 6). At baseline, the majority of ER+/HER2− tumors (56%, 9/16) were immune cold and 78% (7/9) of these cases remained immune cold on-treatment. On the other hand, all TN tumors switched to a more immune stimulated state on-treatment (71%, 17/24) or remained immune hot at both stages (29%, 7/24). Immune state transition is also linked to pathologic response (*p* = 0.097). None of the ten patients with tumors that remained immune cold at T1 and T2 achieved pCR compared to 45.8% (11/24) of the cases that changed to a more immune stimulated state on-treatment.

**Fig. 6:**
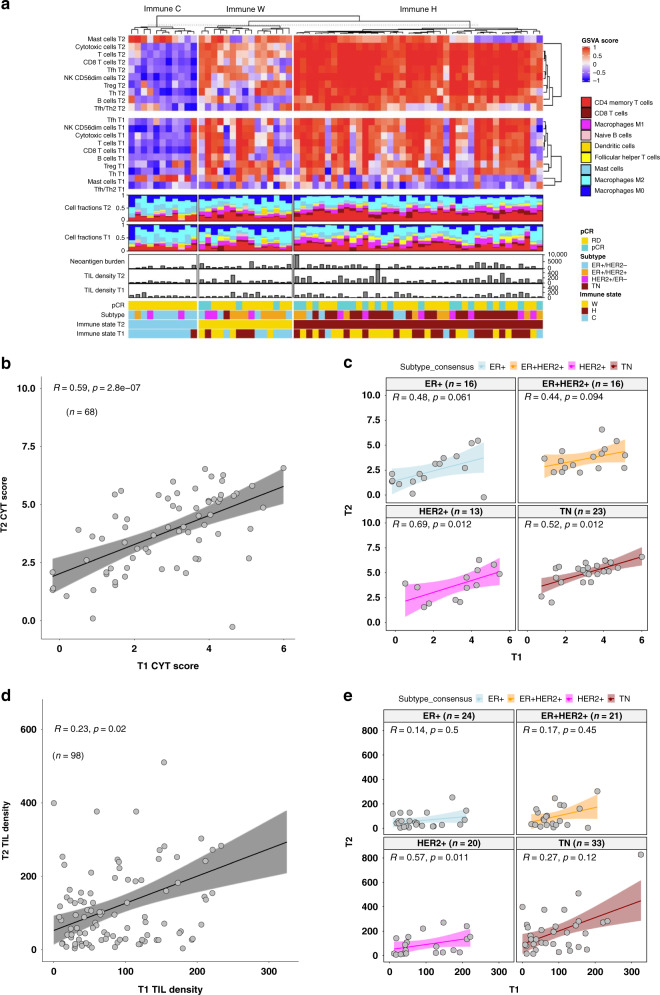
Comparing immune states at on-treatment vs. baseline. **a** Integrated map of immune signatures and immune cell fractions for paired T2 and T1 samples. Immune state classifications were based on T2 samples. **b**, **c** Scatterplots showing CYT scores in paired T1 vs. T2 samples in the overall cohort (**b**) and different subtypes (**c**). Source data are provided as a Source Data file. **d**, **e** Scatterplots showing TIL densities in paired T1 vs. T2 samples in the overall cohort (**d**) and different subtypes (**e**). Statistical significance of correlation was determined using the Spearman method. Shaded error band around the regression line indicates the 95% confidence interval. Source data are provided as a Source Data file.

Despite variability over time, the immune features appeared to be strongly correlated between paired on-treatment and baseline samples with a significant association of the immune states between T1 and T2 (*p* = 7.79e−06) (Fig. 6a and Supplementary Fig. 6f). CYT scores were also significantly correlated between paired on-treatment and baseline samples (rho = 0.59, *p* = 2.8e−07) (Fig. 6b). In addition, CYT scores were positively correlated between T1 and T2 samples in different subtypes with significant correlations in HER2+ (rho=0.69, *p* = 0.012) and TN (rho = 0.52, *p* = 0.012) (Fig. 6c). Consistently, TIL densities in T1 and T2 samples were also positively correlated overall and within subtypes (Fig. 6d, e). Hence the capacity for a tumor to initiate a robust immune response during NAC treatment appears to be predetermined by baseline immune features.

### Virtual microdissection analysis differentiated tissue compartments

We observed lower tumor purity, a measure of the tumor proportions of the bulk tumor, in the immune hot state compared to the other states and during on-treatment compared to the baseline (Supplementary Fig. 7). To address the tumor cellularity as potential confounder in pre-treatment and post-treatment analysis, we included tumor purity as a covariate in our model for differential expression analysis (see “Methods” section). Further, to investigate whether the observed dynamics in immune responses were solely a passenger effect of the tumor compositional changes caused by NAC treatment, we performed virtual microdissection analysis to deconvolute bulk tumor expression profiles into multiple factors analogous to expression signatures that are attributable to distinct tissue compartments of the bulk tumor (see “Methods” section). Besides a composite tumor intrinsic factor, we also identified three factors that represent tumor extrinsic compartments (TME)—tumor infiltrating leukocyte (TIL) factor F13, stromal factor F12, and a composite normal tissue factor by pathway enrichment analyses and comparing factor weight distributions across different breast cancer and normal tissue cohorts (Supplementary Data 5). Tumor purity is positively correlated with the tumor intrinsic factor, and negatively correlated with the TME factors in the overall cohort or within specific subtypes (Supplementary Fig. 8a). Further, the TME factor assignment is supported by patterns of correlation vs. published expression signatures of tumor, stromal, normal tissue and immune cells^34^ (Supplementary Fig. 8b). The TIL factor was strongly correlated with the CYT score as well as various immune signatures. On the other hand, the normal tissue factor was strongly correlated with non-immune signatures derived from blood vessels or normal tissues. The stromal factor was strongly correlated with the Hallmark EMT and TGFβ signatures (Supplementary Fig. 8b, c).

The TIL factor is more significantly enriched in the immune hot state than the normal factor (Kruskal–Wallis test: *p* < 2e−16 vs. *p* = 0.0025), demonstrating that the classification of immune states were driven by changes in the TIL compartment (Supplementary Fig. 9a). All three TME factors increased from T1 to T2 and then decreased from T2 to T3, suggesting that NAC treatment initially induced relative increases in all tumor-extrinsic components that regressed at end-of-treatment (Supplementary Fig. 9b). However, differential distribution of the TIL factor is more significant than the normal or stromal factors (Kruskal–Wallis: *p* = 9.6e−07 vs. *p* = 0.02 and *p* = 1.5e−4). Most importantly, only the TIL factor is significantly associated with pCR at baseline (Wilcoxon: *p* = 0.0024) and on-treatment (Wilcoxon: *p* = 0.00061) with a consistent pattern of association across all subtypes (Supplementary Fig. 10). Hence, treatment-induced immune response appeared likely to exert a functional impact.

### Immune responses predicts clinical outcome

**Fig. 7:**
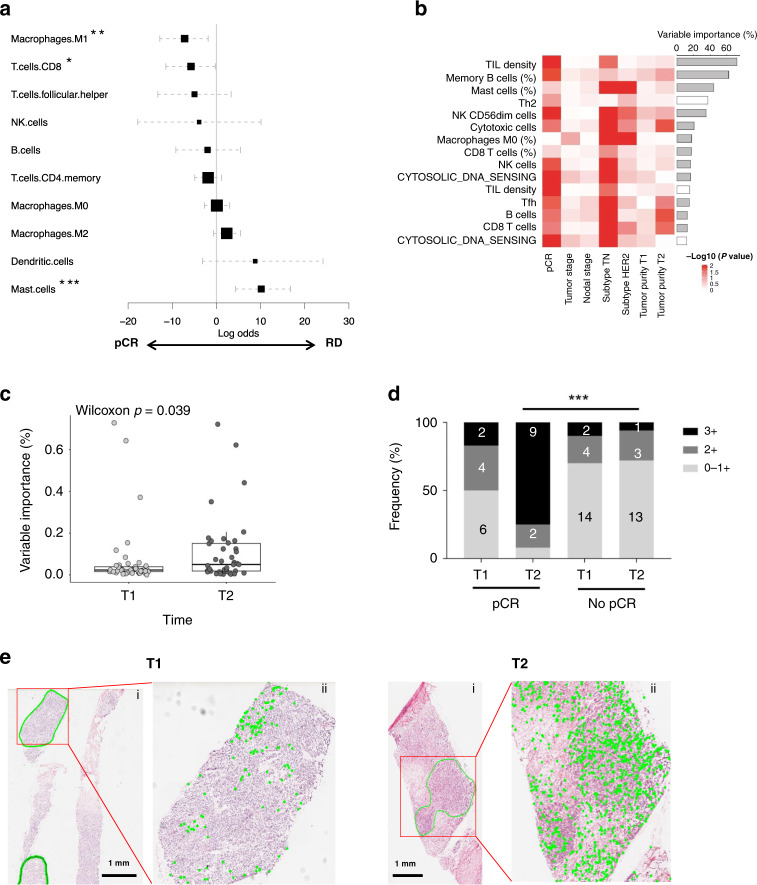
Impact of immune response on treatment outcome. **a** Forest plot showing the associations of different immune cell fractions vs. NAC response combined T1 and T2 samples. The *x*-axis shows the log odds ratio of % cell fractions in pCR vs. RD. Asterisks indicate statistical significance based on multiple regression adjusting for subtype as a covariate: *p = 0.038, **p = 0.008, ***p = 0.0008. **b** Independent associations between clinical factors (columns) and molecular features (rows) associated with pCR. Value in each cell represents –log10(*p*-value) of the association between each feature and clinical factor after adjusting for the confounding effect of other factors. Features were ranked in descending order by % variable usage as determined by bootstrapping analysis, the percentage of runs in which the elastic net model selected the variable to predict pCR status. Source data are provided as a Source Data file. **c** Comparison of variable importance estimated for T1 and T2 immune features based on bootstrapping analyses. Statistical significance was determined using two-sided Wilcoxon rank sum test. The box is bounded by the first and third quartile with a horizontal line at the median and whiskers extend to the maximum and minimum value. Source data are provided as a Source Data file. **d** Distributions of intraepithelial TIL levels between T1 and T2 samples and in pCR vs. RD cohorts. Asterisks indicate statistical significance calculated by using one-sided Chi-squared test: ***p = 4.06E−5. **e** H&E images of tumor biopsy sections taken from the same TNBC patient (OB-16-0006) showing an increased abundance of intraepithelial TILs between T1 baseline and T2 on-treatment. Sub-panel ii shows the tumor region annotated in subpanel i at a higher zoom with the intraepithelial lymphocytes highlighted in green. H&E staining was only performed once on tumor biopsy samples.

We examined whether immune features of tumor biopsies taken at baseline or during the early stages of treatment could predict pathologic response at end-of-treatment. Overall TIL intensity and delta TIL density, the difference in TIL density between T1 and T2, were associated with NAC response (Supplementary Fig. 11a, b). These associations were consistent in different subtypes, with more immunogenic subtypes such as TN and HER2+ exhibiting stronger associations than ER+ subtypes. In the TN subtype, NAC response was significantly associated with delta TIL density (*p* = 0.038) as well as TIL levels at both T1 (*p* = 0.0071) and T2 (*p* = 0.024) (Supplementary Fig. 11c–e). Patients with higher fractions of immune suppressive cell types in baseline and on-treatment tumors such as mast cells and M2 macrophages were more likely to harbor residual diseases. Conversely, patients with baseline and on-treatment tumors harboring higher fractions of immune stimulatory cell types such as CD8+ T cells and M1 macrophages were more likely to achieve pathologic complete response (Fig. 7a). This pattern of association was consistent in different subtypes (Supplementary Fig. 12a–d), and supported by CD8+ IHC analysis on a subset of samples (Supplementary Fig. 12e, f).

Multivariate analyses further revealed that many immune features at baseline and on-treatment were independently associated with pCR status after adjusting for subtype and other clinical features including tumor stage, nodal stage, and tumor purity (Fig. 7b and Supplementary Data 6). In addition, elastic net bootstrap analysis revealed that T2 immune features were more predictive of the pCR status than T1 immune features (Fig. 7c). For example, T2 TIL density was ranked by elastic net bootstrap analysis as the most predictive immune feature while TIL density at T1 was ranked at the eleventh (Fig. 7b and Supplementary Data 7). It has been demonstrated that intraepithelial TILs are localized to tumor parenchyma and initiate cytotoxic reactions only when activated by tumor antigens^35^. We quantified stromal TILs located within intratumoral stromal regions and intraepithelial (IE) TILs located within tumor nests for TN samples through pathologist assessment. While the stromal TILs increased from T1 to T2 regardless of pCR status, IE TILs increased only in the pCR group, revealing a link between the intratumoral localization of TILs and NAC response (*p* = 4.06e−05) (Fig. 7d, e). Taken together, our data suggests that NAC treatment induced robust immune response and increased TIL infiltration into tumor nests in a subset of breast cancers, contributing to antitumor killing and favorable clinical outcome.

## Discussion

Through multidimensional characterization of 281 tumor biopsies taken before, during and after NAC from 146 breast cancer patients, we have shown that NAC induced dynamic changes in the tumor immune microenvironment that varied by breast cancer subtype and pCR status. Tumors from the pCR cohort or the more immunogenic TN subtype exhibited a surge in TIL abundance after only one cycle of chemotherapy followed by a drop to levels below the baseline after a full course of treatment. Among different breast cancer subtypes, TN appears to be the most prone to immune stimulation while ER+/HER2− is the least immunogenic. The NAC induced immune-stimulated states harbored increased abundance of CD4+ and CD8+ T cells and up-regulation of T cell inflamed signatures known to predict responses to checkpoint inhibitors, suggesting that NAC had created a window of opportunity by priming the tumors for responding to IO therapies. During this window, patients with more immune stimulated tumors achieved better outcome than those with more immune suppressed tumors. Both gene expression and histopathology characterization indicated that residual tumors post-NAC tend to be immune suppressed with lower TIL abundance, decreased fractions of immune stimulatory cell types and increased fractions of immune suppressive M2 macrophages compared to on-treatment or baseline tumors. Hence, tumors heavily pretreated by chemotherapies tend to be immune suppressed and impaired in its antitumor cytotoxic activity, consistent with reports that response rates to immune checkpoint inhibitors are better in first-line treatment than in subsequent lines and that prior chemotherapy treatment in the metastatic setting is associated with lower CD8+ TILs^36^.

Recently two Phase III clinical trials have demonstrated that chemotherapy in combination with checkpoint inhibitors significantly increased pCR rate and prolonged progression-free survival in patients with triple negative breast cancers^20,37^. However, there was still limited understanding about the molecular basis of the clinical benefit and whether this benefit would extend to other combinations. Furthermore, how to best combine chemotherapy with immunotherapy in terms of timing and sequencing remains an open question. Our study provides clinically derived molecular evidences that chemotherapies are immune potentiating and underscored the roles of TILs as both effector and marker of the clinical benefit conferred by the combination. Our findings suggest that combination with immunomodulatory therapies is more effective if administered during the early stage of chemotherapy, before the second cycle for instance, rather than after the end of treatment. Moreover, IO combination probably improves outcome only for a subset of patients whose tumors are immunogenic at the outset. Hence, it will become increasingly important in the future to develop therapeutic approaches that reinstate immunological surveillance for cancers that are immune quiescent or immune suppressed in the post-treatment setting.

Most studies focused on characterization of treatment naïve tumors for identifying determinants of tumor immunogenicity and predicting response to IO therapies^38,39^. Evidence is emerging that one can obtain valuable information by characterizing immune response during treatment. It has been reported that increased ratio of CD8+ TILs over FOXP3+ T_REG_ cells after anthracycline-based chemotherapy is predictive of pathologic complete response and survival in breast cancers^40,41^. Another study concluded that on-treatment TILs, but not baseline TILs, are independently associated with pathologic response following chemo-free anti-HER2 therapies in HER2+ breast cancers^42^. In our study, baseline TIL abundance was associated with pCR status consistent with earlier reports^43^. More importantly, we found that immune response quantified by TIL abundance on-treatment is a superior predictor of treatment outcome than baseline TIL abundance. Further, expansion of intraepithelial TILs during early response only among patients who achieved pCR indicates that TILs not only increased in numbers but also mediated anti-tumor activities that led to favorable clinical outcome for those patients. Our observations of breast cancer associated immune states undergoing dramatic changes during neoadjuvant chemotherapy treatment underscored the dynamic nature of tumor-host immune interactions. Immune responses to chemotherapies appeared to be strongly influenced by pre-existing immune features in untreated tumors, indicating that tumor immunogenicity is largely an intrinsic property although the phenotype is more apparent in the on-treatment than pre-treatment tumor. Hence, monitoring tumor immune dynamics over time during chemotherapy and other anticancer treatment could enable early prediction of therapeutic response with better accuracy than baseline characterization alone.

## Methods

### Patient enrollment and sample collection

This study was reviewed and approved by the Institutional Review Board (IRB) of Samsung Medical Center (SMC), Seoul, Korea (IRB No. 2014-11-015) with informed consents from the patients for the research use of clinical and genomic data. Clinical study was registered with www.clinicaltrials.gov (NCT02591966). All patients were diagnosed with histologically confirmed invasive breast cancer and treated at SMC. Patients were treated with a standard neoadjuvant chemotherapy [AC-D(H)] protocol of four cycles of doxorubicin plus cyclophosphamide (AC) combination chemotherapies followed by four cycles of docetaxel (T) chemotherapy. In accordance with ASCO guidelines, HER2+ BC patients were treated with docetaxel plus trastuzumab after AC. For each patient, tumor core biopsy and matched blood were prospectively taken before treatment (T1). A second core biopsy was taken three weeks after the first cycle of AC (T2) while a third tumor sample was taken at surgery following 6 months of treatment (T3).

Fresh frozen tissue specimens were collected from 201 of the 210 recruited patients. All the biopsy samples were processed immediately or within 15 min after acquisitions. Samples at surgery (T3) were grossly examined and taken at the operating table just after the removal of the specimen. We excluded specimens that were invisible, had very small, scattered tumors or contained mostly necrotic or fibrotic scar-like tissue at the time of gross examination. Most of the samples after breast conserving surgeries were sent for specimen mammography to ensure the clips were located at the central area of the tumors before the neoadjuvant chemotherapy according to the protocol of SMC.

### Tumor purity estimates

H&E slides of 5-μm thickness were prepared and analyzed by two pathologists (YLC, SYC) to determine the presence and percentage of tumor cells. Tumor-rich areas were marked for manual macro-dissection whenever necessary and large areas of necrosis were avoided. Tumor purity was estimated as the percentage of tumor cells among all cells (tumor cells, lymphocytes, and normal cells) in the marked tumor area by microscopy. We excluded 55 cases from next-generation sequencing (NGS) due to low tumor purity and low DNA/RNA yield or quality (Fig. 1b). We also computationally inferred tumor purity using FACETS^44^ (Supplementary Data 1) and excluded five samples from downstream analyses with low tumor purity (<20%) based on both computational and pathologist estimates. NGS data QC analyses excluded another four samples due to insufficient read coverage or an outlier expression pattern. FACETS derived tumor purity estimates were used as covariates in regression analyses.

### Whole transcriptome sequencing

For RNA-Seq, sequencing libraries were prepared using TruSeq RNA Sample Preparation kit v2 (RS-122-2001 and RS-122-2002, Illumina). Sequencing of the RNA libraries was performed on an Illumina HiSeq2500 in 100-bp paired-end mode of the TruSeq Rapid PE Cluster kit and the TruSeq Rapid SBS kit. RNA-Seq was analyzed using the RSEM^45^ pipeline with hg19 as the genome reference.

### Breast cancer subtype classification

The IHC subtypes were determined by IHC assays for ER, PR, and HER2 and used in clinical diagnoses and treatment. For confirmation, we also predicted PAM50 subtypes using Genefu^46^ from the gene expression data. The following rules were used to map IHC subtypes with PAM50 subtypes: ER+ (Luminal A and Luminal B), ER+/HER2+ (Luminal B and Her2), HER2+ (Her2) and TN (Basal). In cases of disagreement between IHC and PAM50, we chose a subtype by examining individual markers including *ERBB2*, *ESR1*, and *PGR* gene expression and *ERBB2* copy number^47^.

### Differential expression and pathway analysis

We applied linear mixed effects model to identify genes differentially expressed over NAC treatment time points while adjusting for the confounding effects of breast cancer subtypes and tumor purity. DE analyses were performed separately for three pairwise comparisons (T1 vs. T2, T2 vs. T3, and T1 vs. T3) in different sample groups, including the overall cohort and three subtypes—ER+, HER2+ and TN. The ER+ /HER2+ subtype was excluded due to insufficient sample size at T3. An aggregate list of significant DE genes were selected by requiring *p*-value < 0.01 and absolute fold-change > 2 from all pairwise comparisons and sample groups. K-means based consensus clustering^48^ was performed on expression profiles of the significant DE genes from each sample group to identify three clusters per group.

Hypergeometric tests were performed on each cluster of significantly DE genes to identify enrichment with known cancer related pathways, derived from three collections of curated gene sets from MSigDB v5.1^49^—HALLMARK, KEGG, and REACTOME. We calculated gene expression signature scores for curated MSigDB pathways and major immune and tumor associated cell types^34^ using the GSVA algorithm^28^. A pathway was mapped to a DE gene cluster if it is enriched with FDR < 20% and has a differentially expressed expression signature with a FDR < 5% in the same sample group for at least one pairwise comparison (T1 vs. T2, T2 vs. T3, and T1 vs. T3). To further resolve immune cell mixtures and discriminate closely related cell types, we implemented an in silico immune cell deconvolution using a nu-support vector regression (nuSVR) method^32^ to infer the relative fractions of 13 immune cell subtypes among all leukocytes present in each tumor using RNA-Seq data.

### Identifying differentially distributed features over time

The distributions of molecular features including voom^50^ normalized gene expression, gene signatures, and inferred immune cell fractions were compared across different treatment times to identify significant differences. Let *ω*_j_ represent the data set containing the random variable observations *y*_ij_ for feature *j* along with clinical information (_*pi*_, *s*_i_, *t*_i_, *d*_i_, and *r*_i_) for the *N* samples (*i*), where feature *y*_ij_ information was available, such that $\omega _{\mathrm{j}}\,=\,\{ y_{{\mathrm{ij}}},p_{\mathrm{i}},s_{\mathrm{i}},t_{\mathrm{i}},d_{\mathrm{i}},r_{\mathrm{i}}\} _{i = 1, \ldots ,N}$. Since features are assumed to be mutually independent and treated in a univariate fashion, the feature index *j* is omitted further in this section for simplicity. Clinical information includes sample tumor purity (*p*), which describes percent of tumor tissue present in a given sample, consensus subtype (*s*), which lists the modal tumor subtype per patient, collection time (*t*), which represents the treatment time each sample was obtained, the patient donor (*d*) for each sample, and the pathologic response status (*r*) for the donor patient.

NAC induced feature changes over time were modeled using linear mixed-effects regression model such as

1 $$y_{\mathrm{i}} = \beta _0 + \beta _1p_{\mathrm{i}} + \beta _2s_{\mathrm{i}} + \beta _3t_{\mathrm{i}} + b_{\mathrm{d}} + \varepsilon _{\mathrm{i}},$$

where *β*_0_ is the overall feature value (intercept), *β*_1_ is the tumor purity effect on feature values, *β*_2_ estimates the feature differences due to subtypes, *β*_3_ describes the treatment time effects on feature values, *b*_d_ is a normally distributed random variable with mean zero representing the deviation from the overall mean of the mean feature value for the *d*th donor patient (between-patient residuals), and *ε*_i_ is a normally distributed random variable with mean zero accounting for the within-patient residuals. The reduction in the residual sum of squares given by *β*_3_ was assessed by Chi-squared tests and features were retained for further investigation, if the addition of the time covariate was shown to be statistically relevant for the model goodness-of-fit (FDR^51^ < 0.05). A similar approach was applied to identify subtype specific NAC induced feature changes over time but now with the exclusion of the term *β*_2_*s*_i_ from Eq. (1). The *lmerTest* R package^52^ was used for these analysis.

### Multiplexed immunofluorescence (IF) and immunohistochemistry (IHC)

Multiplexed IF staining was performed using the Opal 7 Solid Tumor Immunology Kit (PerkinElmer). According to the manufacturer’s protocol, formalin-fixed paraffin-embedded tissue slides were deparaffinized with xylene and rehydrated through an ethanol gradient ending with a distilled water wash. Antigen retrieval was performed using AR9 (for PD-L1, CD4, and CD8) or AR6 (for CD45RO, CD3, and pan-cytokeratin) buffer and microwave treatment. The first antibody PD-L1 (clone E1L3N) was incubated, followed by secondary antibody incubation using Opal Polymer HRP. Opal-620 dye was then applied for visualization of PD-L1, and microwave treatment was performed to remove primary and secondary antibodies. The process was repeated with antibodies/fluorescent dye in the following order: CD4 (clone EP204)/Opal-520, CD8 (clone 4B11)/Opal-570, CD45RO (clone UCHL1)/Opal-650, CD3/Opal-540, pan-cytokeratin (clone AE1/AE3)/Opal-690. After applying DAPI for visualization of nuclei, slides were mounted, and cover slipped. Multiplexed slides were imaged using PerkinElmer Vectra Polaris quantitative slide scanner, and images were analyzed using Inform software (PerkinElmer, Ver. 2.4.1). Immunohistochemistry for CD8 was conducted in FFPE tissues using a CONFIRM-anti-CD8 (SP57) rabbit monoclonal primary antibody without dilution with Ventana BenchMark XT via the OptiView DAB IHC Detection Kit.

### TIL quantification based on digital H&E image analysis

Using CRImage^53^, a pathologist marked 273 cells as lymphocytes and 189 cells as non-lymphocytes from H&E diagnostic breast cancer tiles from Samsung Medical Center at 20× magnification. Parameters used for segmentation were otsu threshold, minShape = 50, failureRegion = 2000, and maxShape = 800. CRImage generates 36 parameters using EBImage^54^, which were used to train the support vector machine classifier to label the cells and output coordinates and features of each cell. The SVM model was generated by the e1071 svm function with parameters type = “C”, kernel = “radial”, probability = TRUE. Using a Wilcoxon rank sum test on each separate parameter, 30/36 parameters had a *p*-value < 1e−05 between lymphocyte and non-lymphocyte classes showing that the univariate parameters were very significant. We tiled 20× H&E images into 2050 × 2050 pixel tiles using “vips dzsave”. Tiles were segmented with the same parameters as the training set and classified with the SVM model. Output files for each tile were consolidated to reconstruct the global coordinates of the features by calculating the offset from the tile’s relative position and pixel size. The SVM generated a score from 0 to 1 to indicate confidence in each class. Cells labeled lymphocytes were filtered to be 60–150 in size and have a minimum SVM score of 0.97. Cells labeled non-lymphocytes had a SVM score ≤ 0.1. Large cells were defined as cells not labeled lymphocyte and having a size ≥ the third quartile of all cells >200 in size. The tissue areas from H&E images were calculated using Visiopharm^TM^ (Visiopharm, Hoersholm, Denmark) using a custom algorithm. The lymphocyte density from H&E images was then calculated by dividing the lymphocyte count obtained from CRImage by the estimated tissue area for each patient. The data was fit with a linear model and R2 calculated using lm.

### Immune state classification

We used iCluster^55^ to cluster and classify samples as a joint multivariable regression of multiple data types including immune gene expression and immune cell fractions with reference to a set of common latent variables that represent the underlying immune states. The optimal number of clusters was determined based on Bayesian information criterion. The association of immune states with immune features (*Y*_i_) was calculated using ANOVA by solving the following equation:

$$Y_{\mathrm{i}} = \beta _0 + \beta _1p_{\mathrm{i}} + \beta _2s_{\mathrm{i}} + {\it{\epsilon }}_{\mathrm{i}},$$

where *β*_0_ is the overall feature value (intercept), *β*_1_ is the tumor purity effect on feature values, *β*_2_ estimates the feature differences due to immune states. The lm function from the stats R package was used to estimate the coefficients.

### Virtual microdissection analysis

We performed virtual microdissection analysis using the non-negative matrix factorization (NMF) algorithm to separate bulk tumor expression into factors representing distinct tissue compartments^56^. NMF was applied on a compendium of RNA-Seq gene expression that included the current cohort (NAC) and 1678 samples from TCGA, CCLE, GTEx, and the SMC YBC cohorts^47^. The NMF algorithm factorizes the gene expression matrix V of *g* genes and *s* samples into two non-negative matrixes of *k* factors: gene factor matrix W of *n* gene weights for *k* factors and sample factor matrix H of *m* sample weights for *k* factors. W represents the expression pattern of the *k* parts and H represents the respective contribution of *k* parts in each sample or bulk tumor^57^. NMF was performed on log transformed gene expression matrix V, log_2_(TPM+1), of the combined cohorts using the R package NMF which used the “brunet” algorithm^58^. We performed 30 runs of NMF and chose the factorization that achieved the lowest approximation error for subsequent analyses. To extract exemplar genes for each of the *k* factors, a score for each gene *g* was first calculated representing how factor-specific it is based on an entropy measure^59^. Two criteria were then used for selecting the genes. First, the gene score has to be greater than $\bar \mu + 3\bar \sigma$, where $\bar \mu$ and $\bar \sigma$ represents the median and the median absolute deviation (MAD) of the scores respectively. Second, the maximum contribution to a basis component of the feature has to be greater than the median of all contributions. Pathway enrichment analyses were performed on the exemplar genes for each factor using the Fisher’s exact test and the MSigDB v5.2 pathway gene sets. To determine the optimal *k*, we compute the cophenetic coefficient and choose *k* = 14 that maximize the coefficient score^58^. To attribute NMF factors to different tissue compartments, we examined the distribution of sample factor weights in sample groups with known labels and examined the enriched pathways based on the exemplar genes. We identified four tumor intrinsic factors including F14 for the TN subtype, F2 for HER2+, and two ER+ factors—F1 and F4. We also identified four factors that represent tumor extrinsic compartments (TME)–TIL factor F13, stromal factor F12 and two normal tissue factors F3 and F7. F3 was overweight in tumor adjacent tissue while F7 was more enriched in healthy normal tissue from the GTEx study. Tumor intrinsic and normal tissue factor weights were summed to create two composite factors—F-Tumor and F-Normal.

### Multivariate analysis

We performed multiple regression analyses with adjustment for confounder variables to assess the associations between immune features and clinical features. Seven clinical variables were evaluated—pathologic response status (pCR vs. RD), subtype TN (yes vs. no), subtype HER2+ (yes vs. no), tumor stage (early vs. late), nodal stage (early vs. late), and tumor purities at T1 and T2. Three types of immune features from tumor biopsies taken at T1 and T2 were evaluated: histopathology analysis (TIL density), immune cell fractions (e.g., % CD8+ T cells, % CD4+ memory T cells, % Mast cells) and gene expression signatures (e.g., cytotoxic cells, NK cells, and CD8+ T cells). Logistic regression was applied if the immune feature was a binary variable and regular linear regression was applied for continuous variables. For multiple linear regression analyses, we solved the function $Y = \beta _0 + \beta _1X_1 + \beta _2X_2 + \ldots + \beta _{\mathrm{p}}X_{\mathrm{p}} + \varepsilon$ where *β*_j_ quantify the association between variable *j* with the response. R function “lm” from “stats” package was used to estimate the regression coefficients *β*_0_, *β*_1_,… *β*_p_ and corresponding *p* values. For logistic regression analyses, we solved the function${\mathrm{log}}( \frac{{p( x )}}{( {1 - p( x )} )} ) = \beta _0 + \beta _1X_1 + \beta _2X_2 + \ldots + \beta _{\mathrm{p}}X_{\mathrm{p}} + \varepsilon$, where $p\left( x \right) = {\mathrm{Pr}}(Y = 1|X)$ and *Y* was the binary response variable. R function “glm” from “stats” package was used to estimate the regression coefficients *β*_0_, *β*_1_,… *β*_p_ and corresponding *p* values.

Elastic Net^60^ is a penalized regression approach to variable selection that identifies linear combinations of unique variables that contribute to a response variable such as pCR status. Elastic Net performs variable selection by minimizing a regularized cost function. Immune features subject to multivariate analysis includes 24 bindea immune signatures^34^ and estimated cell fractions for 13 immune cell types. Features were ranked based on the number of times each feature was selected over 10,000 bootstrap iterations using a penalty factor of 1.

### Reporting summary

Further information on research design is available in the Nature Research Reporting Summary linked to this article.

## Supplementary information

Supplementary Information

Descriptions of Additional Supplementary Files

Supplementary Dataset 1

Supplementary Dataset 2

Supplementary Dataset 3

Supplementary Dataset 4

Supplementary Dataset 5

Supplementary Dataset 6

Supplementary Dataset 7

Reporting Summary

## Data Availability

The RNA-Seq based gene expression data are deposited in GEO under accession code GSE123845. The RNA-Seq raw data generated in this study have been deposited in the European Genome-phenome Archive (EGA) under the accession code EGAS00001003354. The raw data is available under controlled access. Data access can be obtained by contacting Dr. Woong-Yang Park [woongyang.park@samsung.com]. The remaining data are available within the Article, Supplementary Information or available from the authors upon request. Source data are provided with this paper.

## Source data

Source data
