# Supplementary material for: Chemotherapy induces dynamic immune responses in breast cancers that impact treatment outcome
Source: Nat Commun. 2020 Dec 2;11:6175. doi: 10.1038/s41467-020-19933-0 (PMC7710739; doi:10.1038/s41467-020-19933-0)
Supplement: Supplementary file 1 — Supplementary Information [file 41467_2020_19933_MOESM1_ESM.pdf]

Supplementary Figure 1. Distinct clusters of differential expression patterns

a

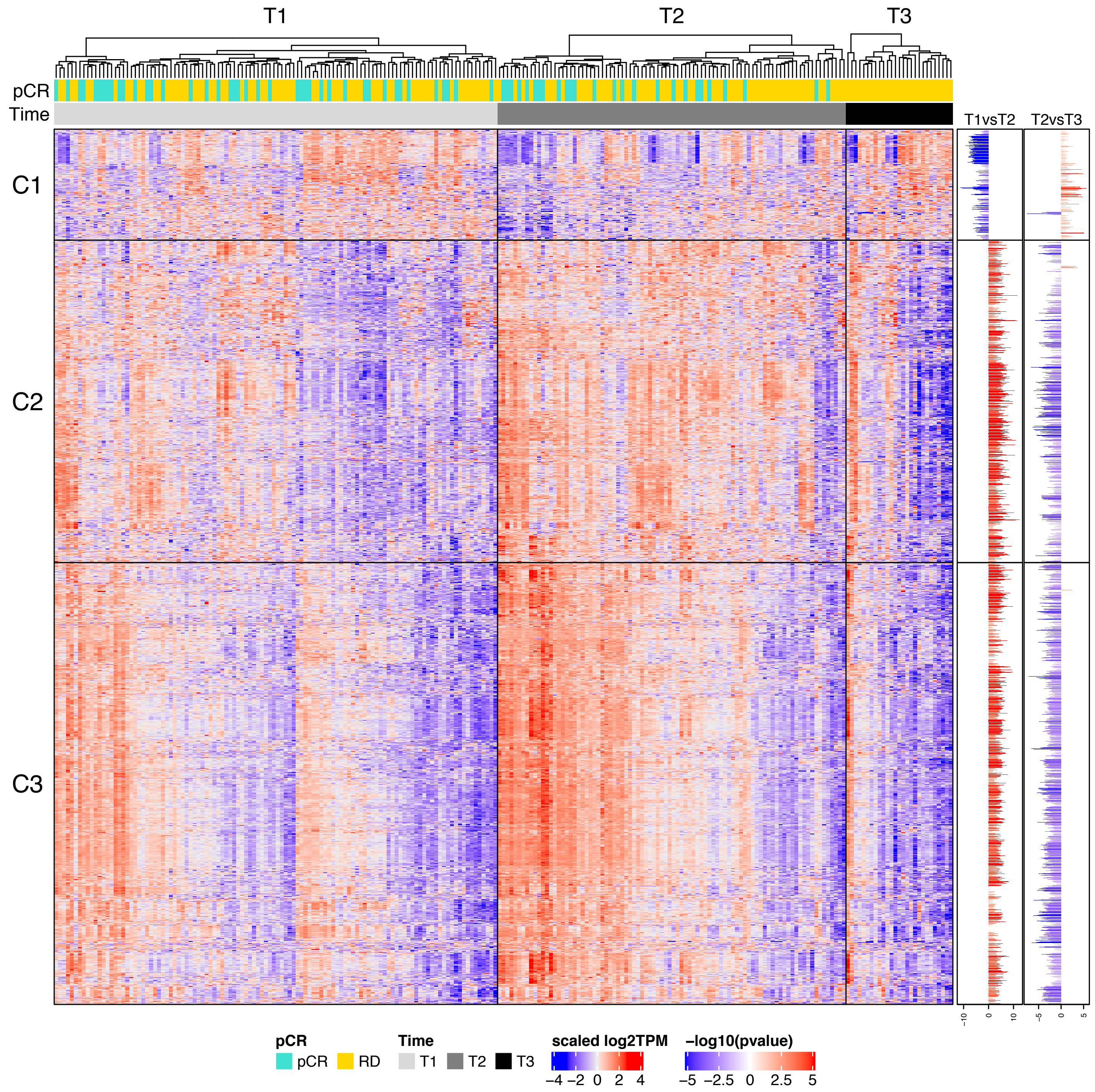

Supplementary Figure 1. Distinct clusters of differential expression patterns

b

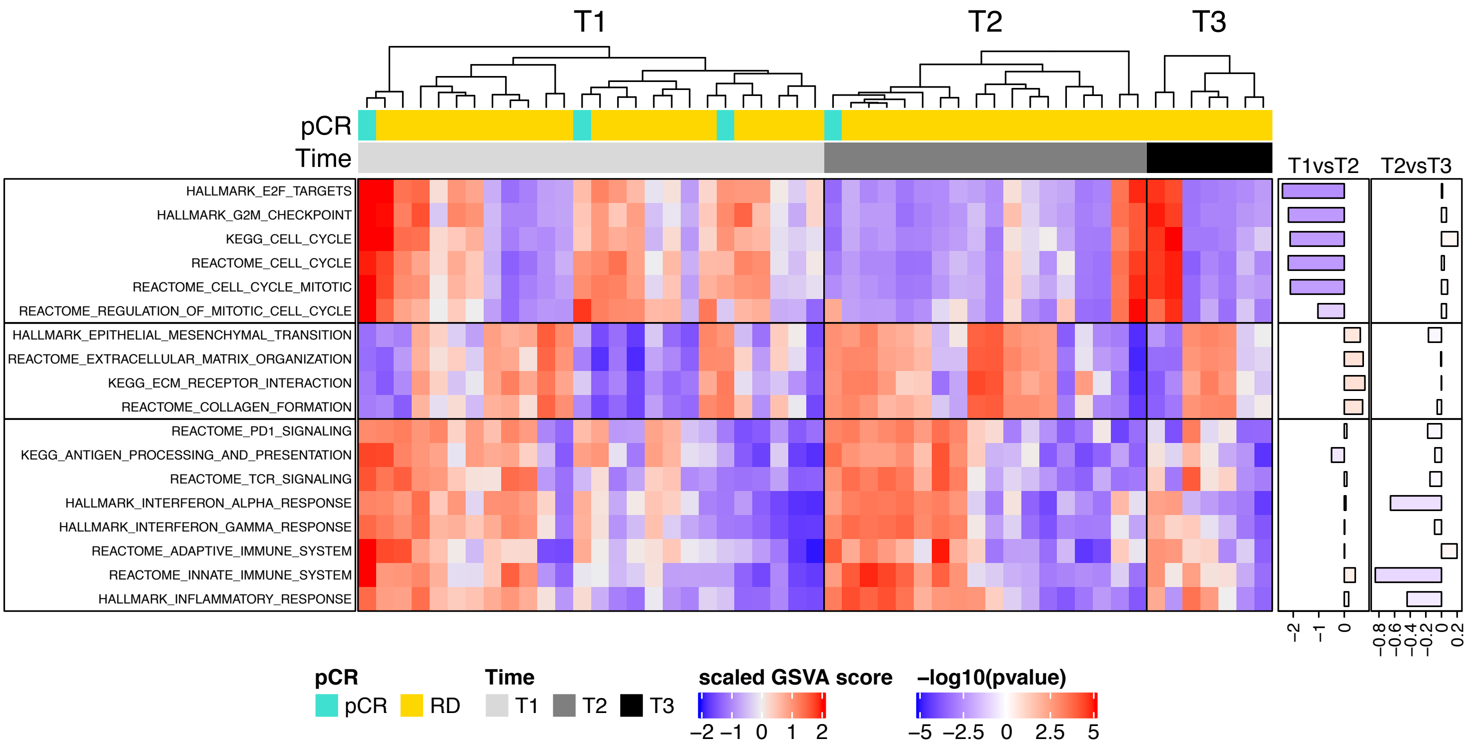

c

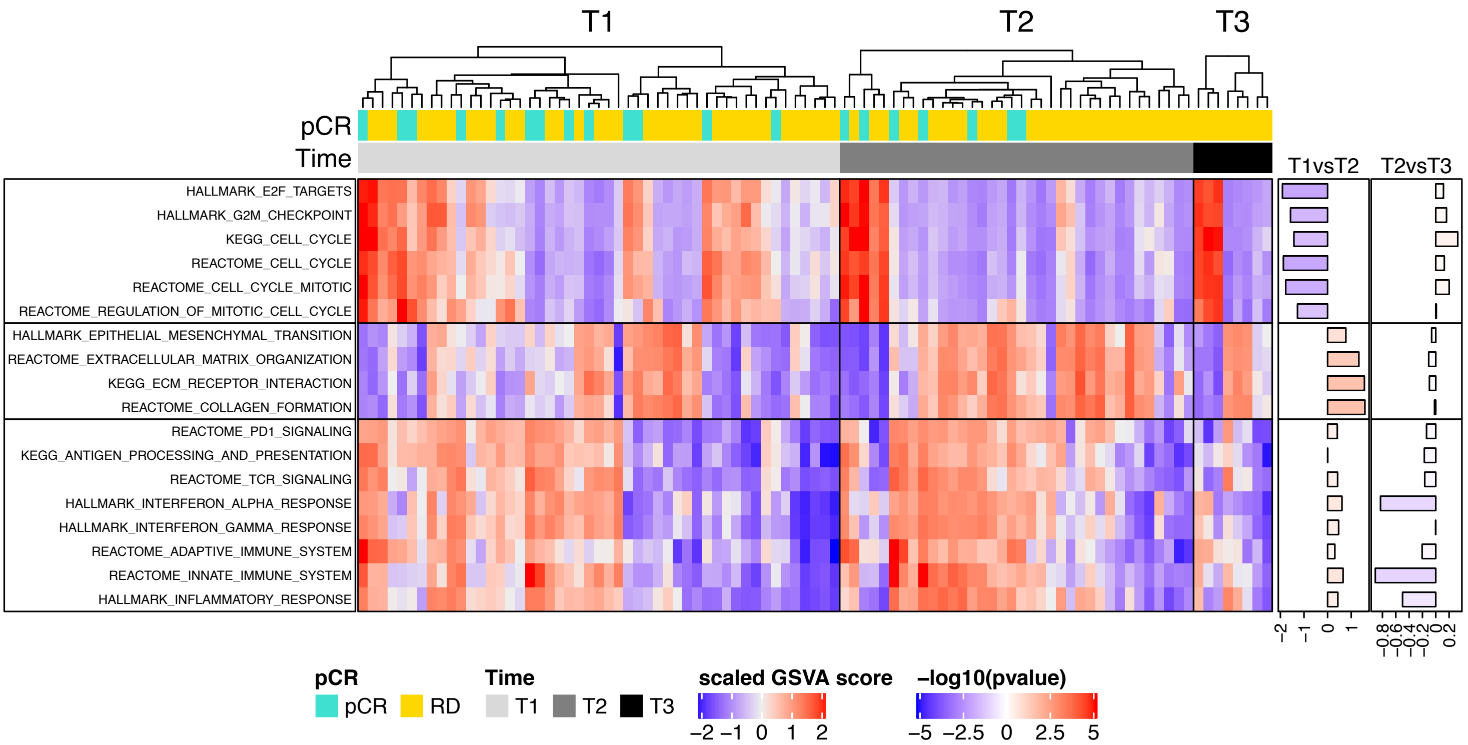

Supplementary Figure 1. Distinct clusters of differential expression patterns

d

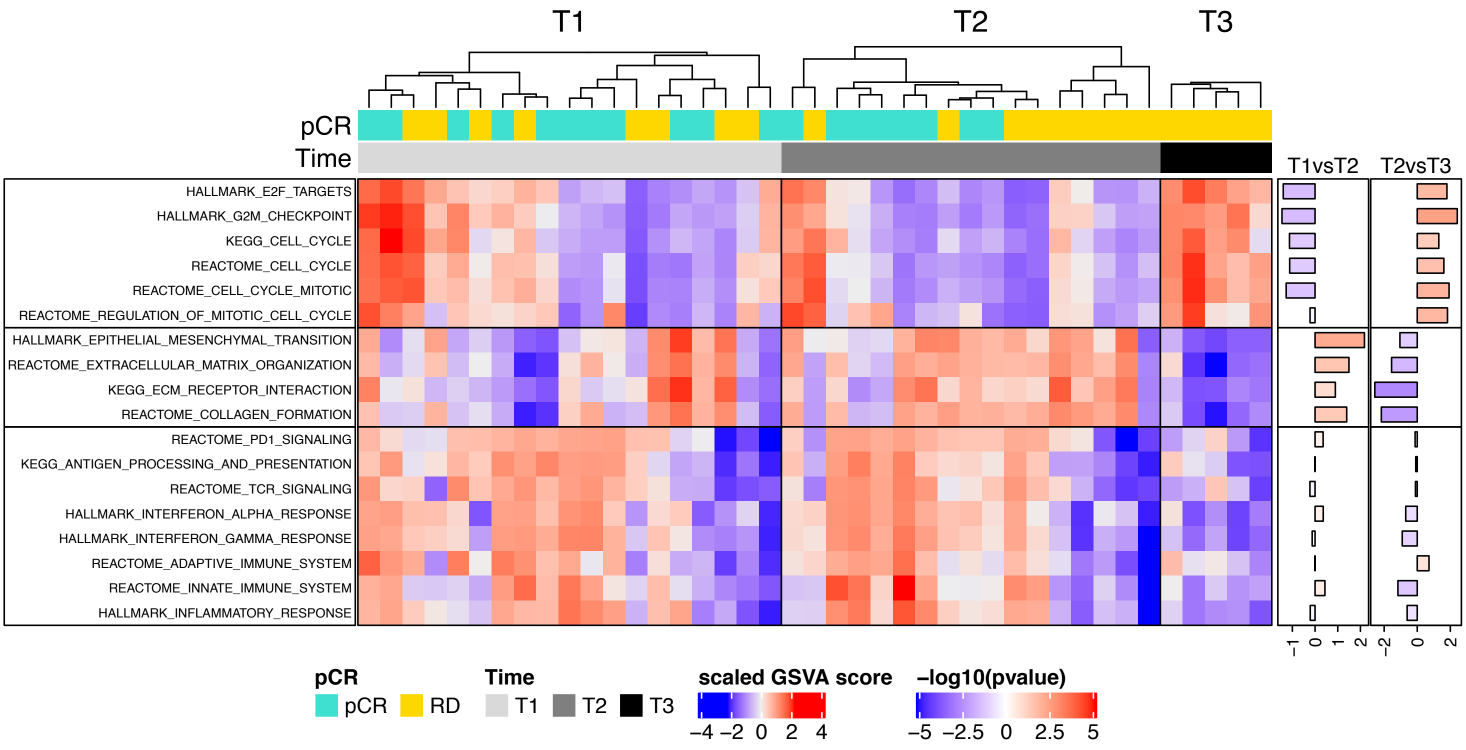

e

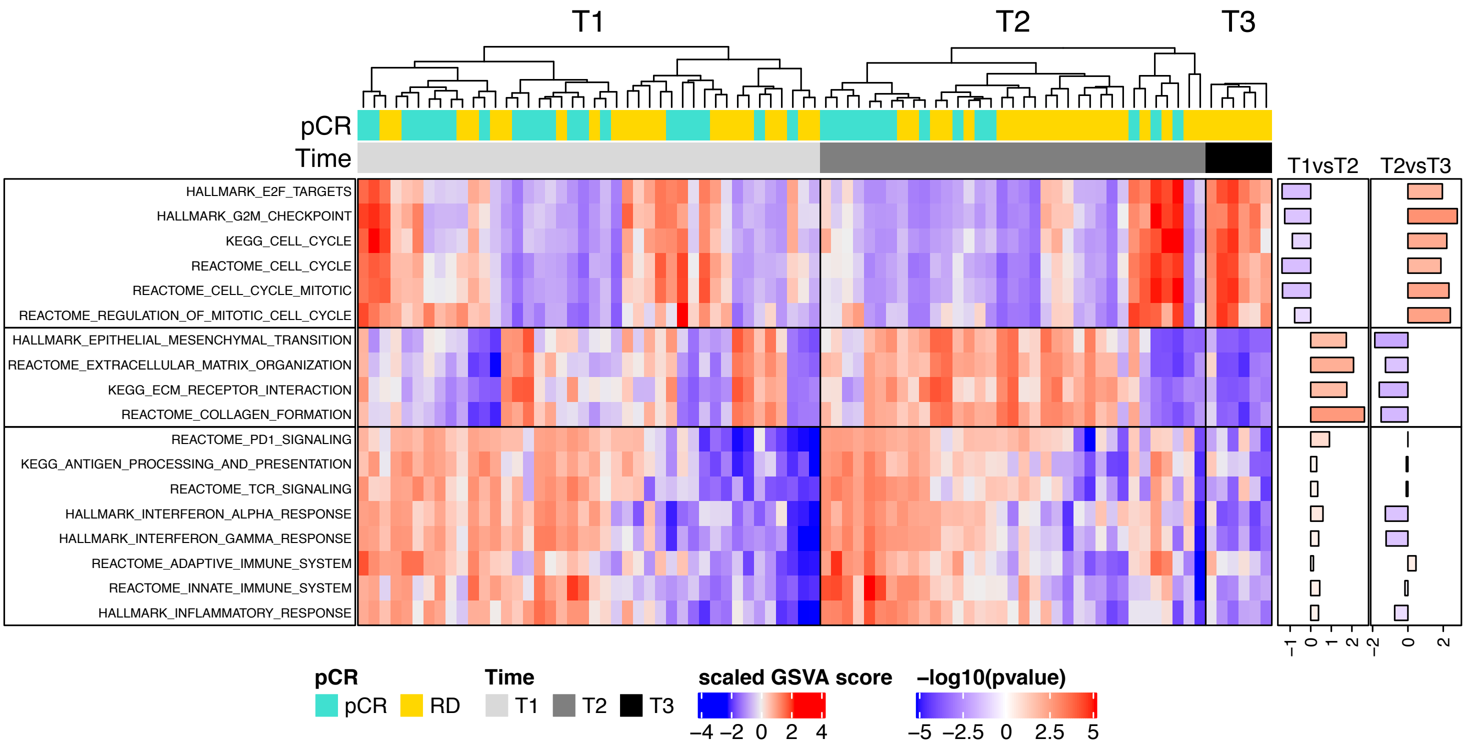

Supplementary Figure 1. Distinct clusters of differential expression patterns

f

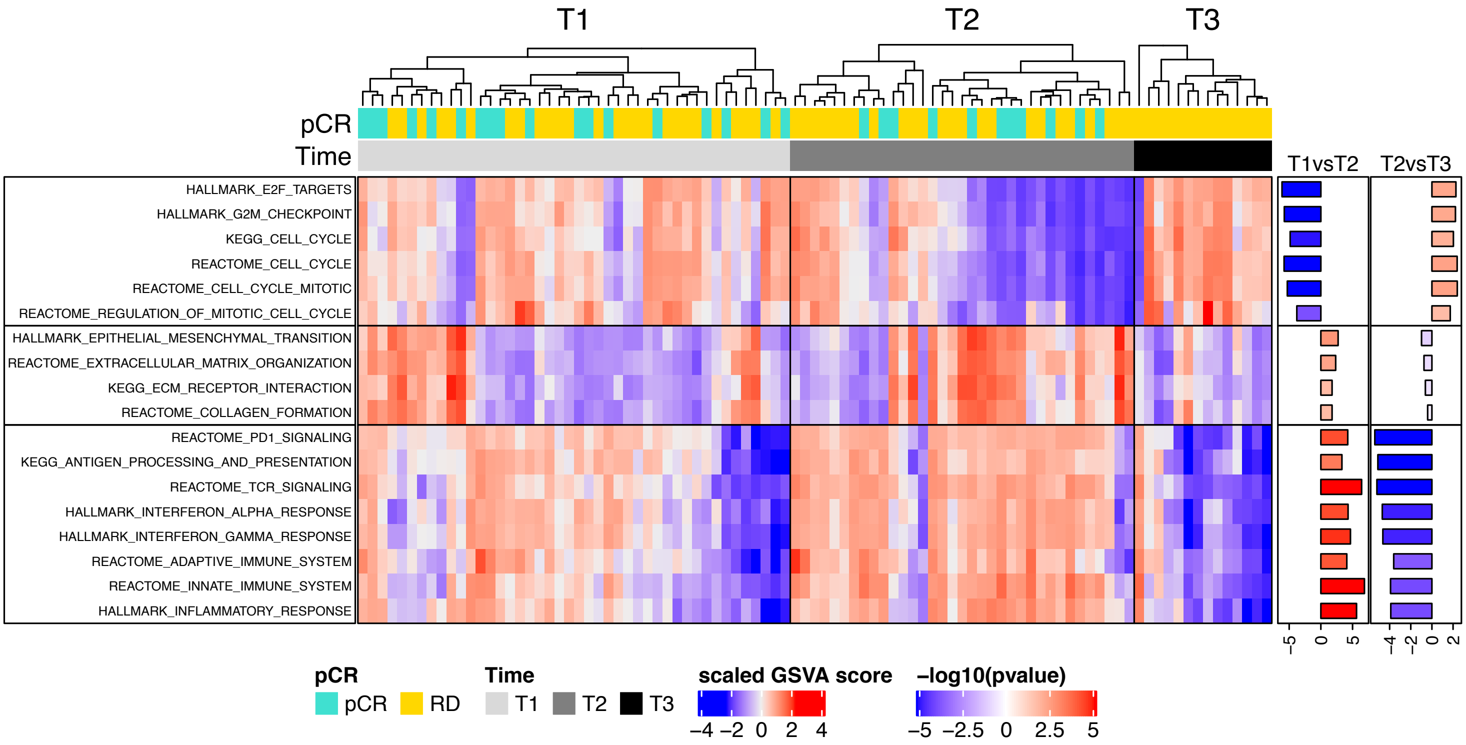

(a) DE genes form three clusters of distinct expression patterns, revealed by pathway enrichment analysis to be enriched in cell cycle (C1), EMT/ECM (C2) and immune pathways (C3). Gene expression in log2(TPM) is shown as centered and scaled z-scores for each gene. Statistical significance of DE was determined using linear mixed-effects regression analysis (LMER) and shown by bar plots on the right as sign*-log10(p-value) based on the sign of the t-statistics. Expression signatures of known pathways corresponding to three cancer hallmarks – cell cycle, immune and EMT in ER+ (b), ER+ combined (c), HER2+ (d), HER2+ combined (e) and TN (f) subtypes. GSVA scores were centered and scaled to z-scores for each pathway geneset. Statistical significance of DE was determined using LMER and shown by the bar plots on the right as sign*-log10(p-value) based on the sign of the t-statistics. Source data are provided as a Source Data file.

Supplementary Figure 2. DE gene patterns in breast cancer subtypes

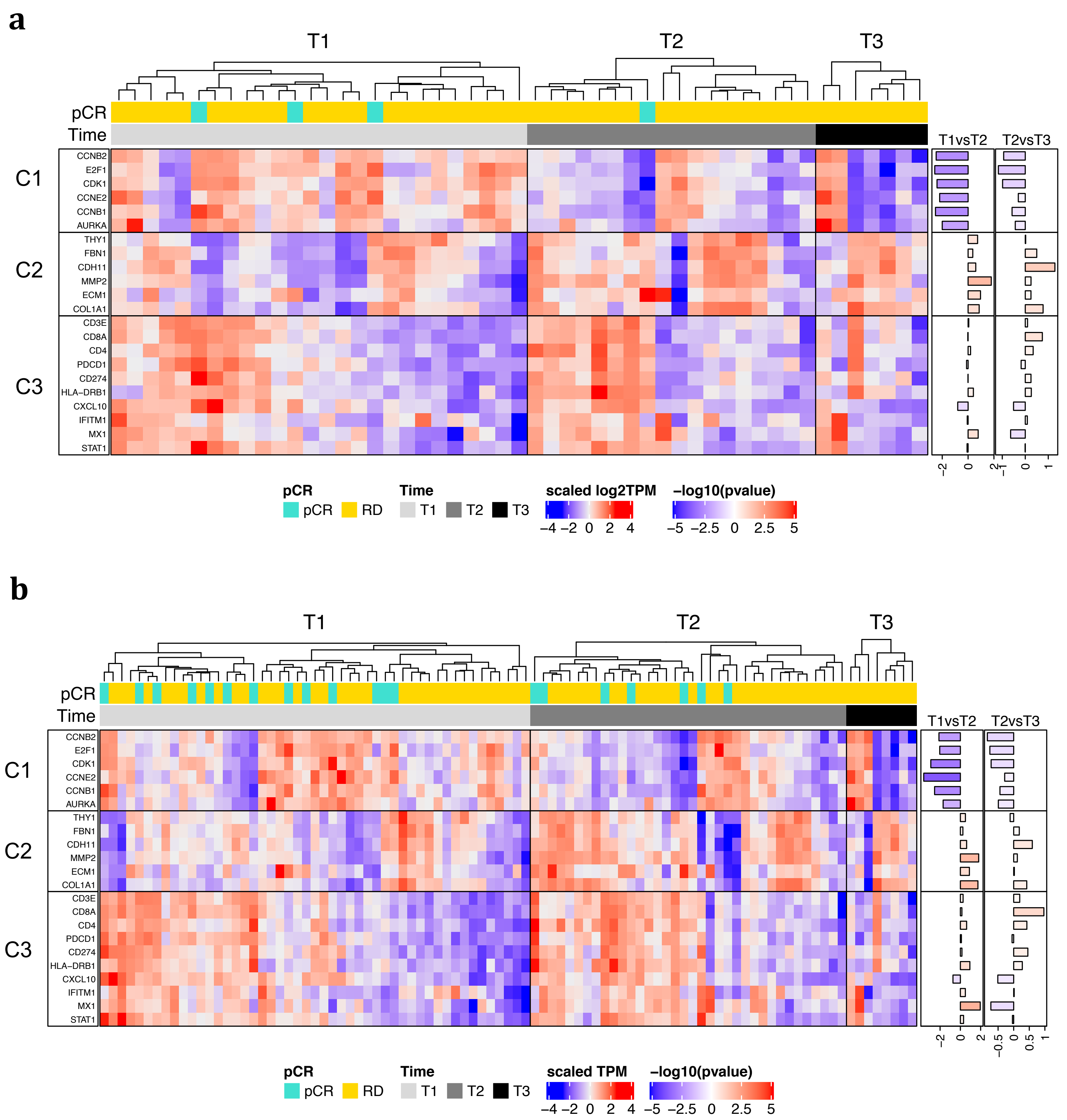

Supplementary Figure 2. DE gene patterns in breast cancer subtypes

c

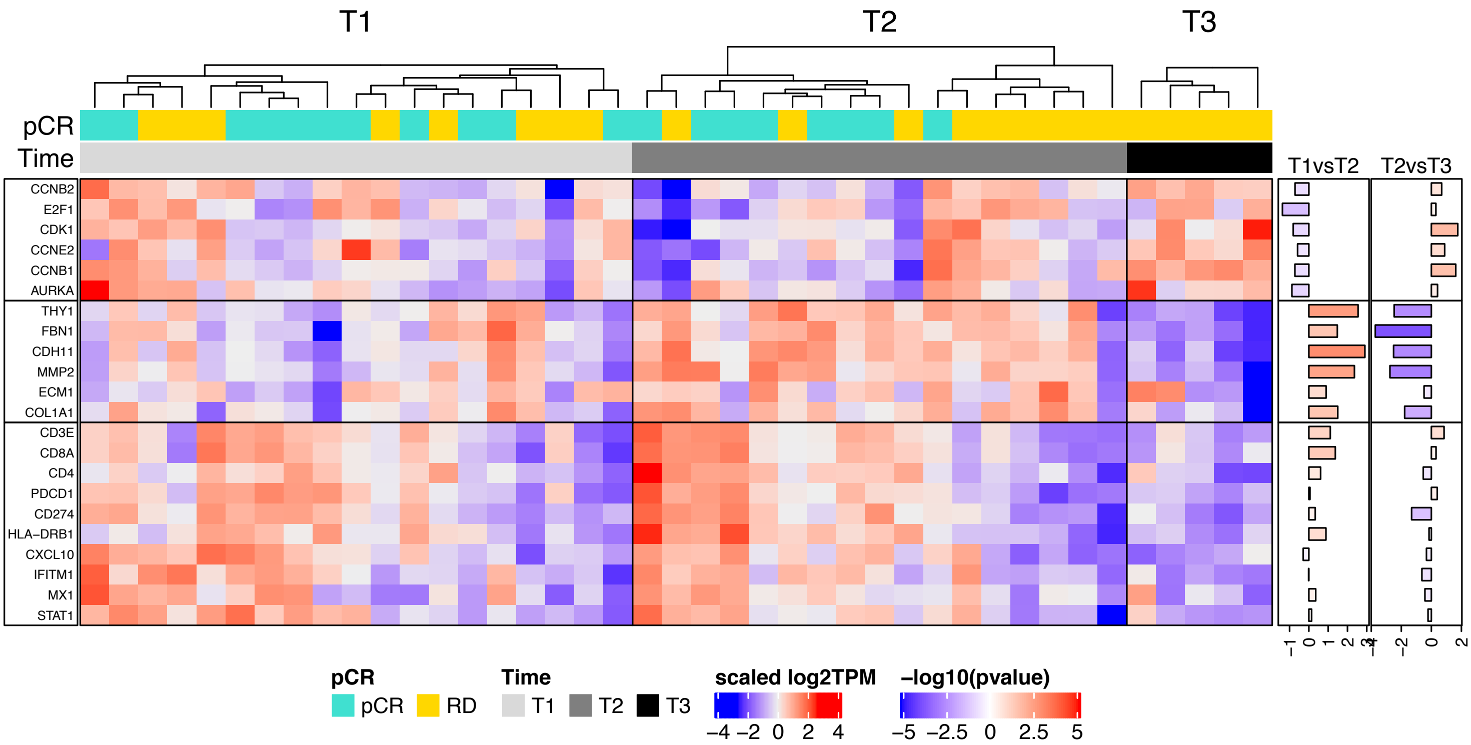

d

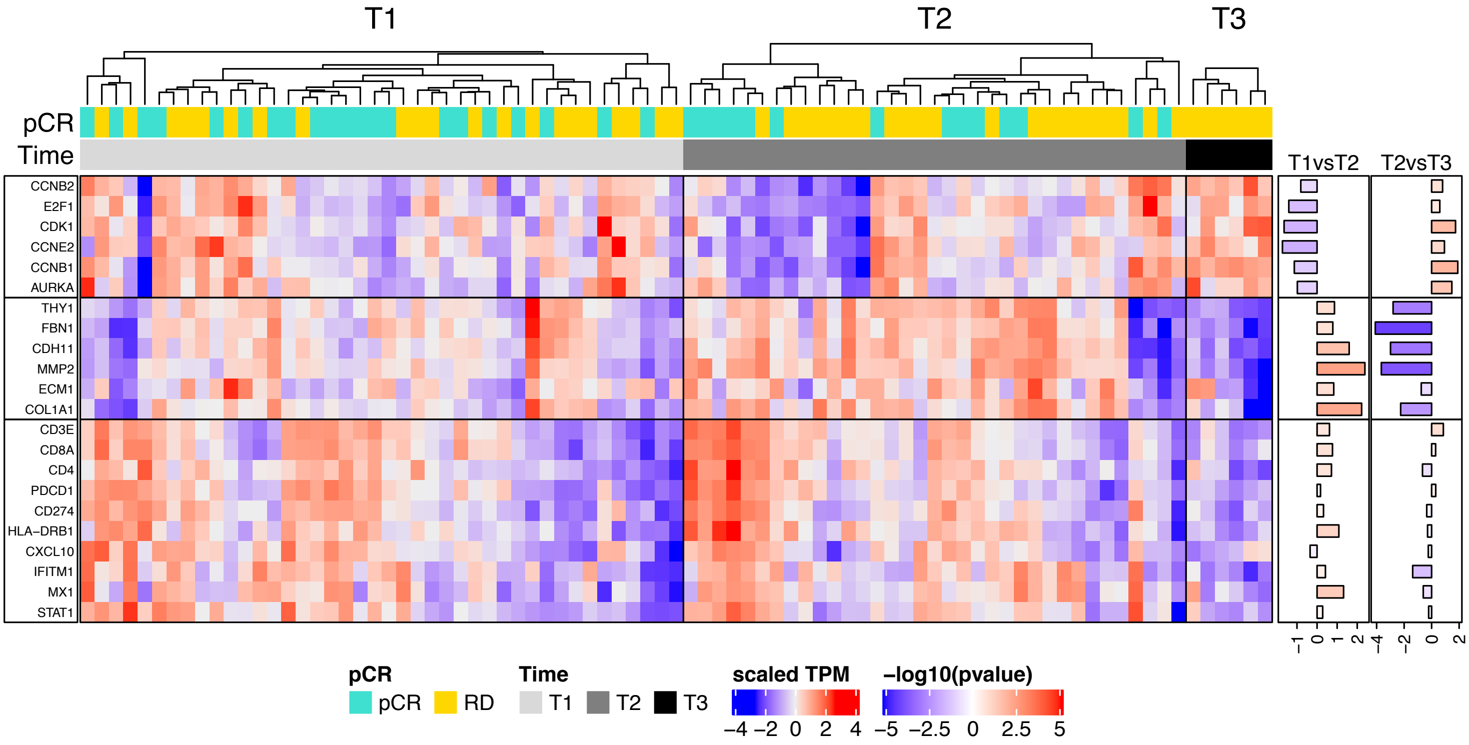

Supplementary Figure 2. DE gene patterns in breast cancer subtypes

e

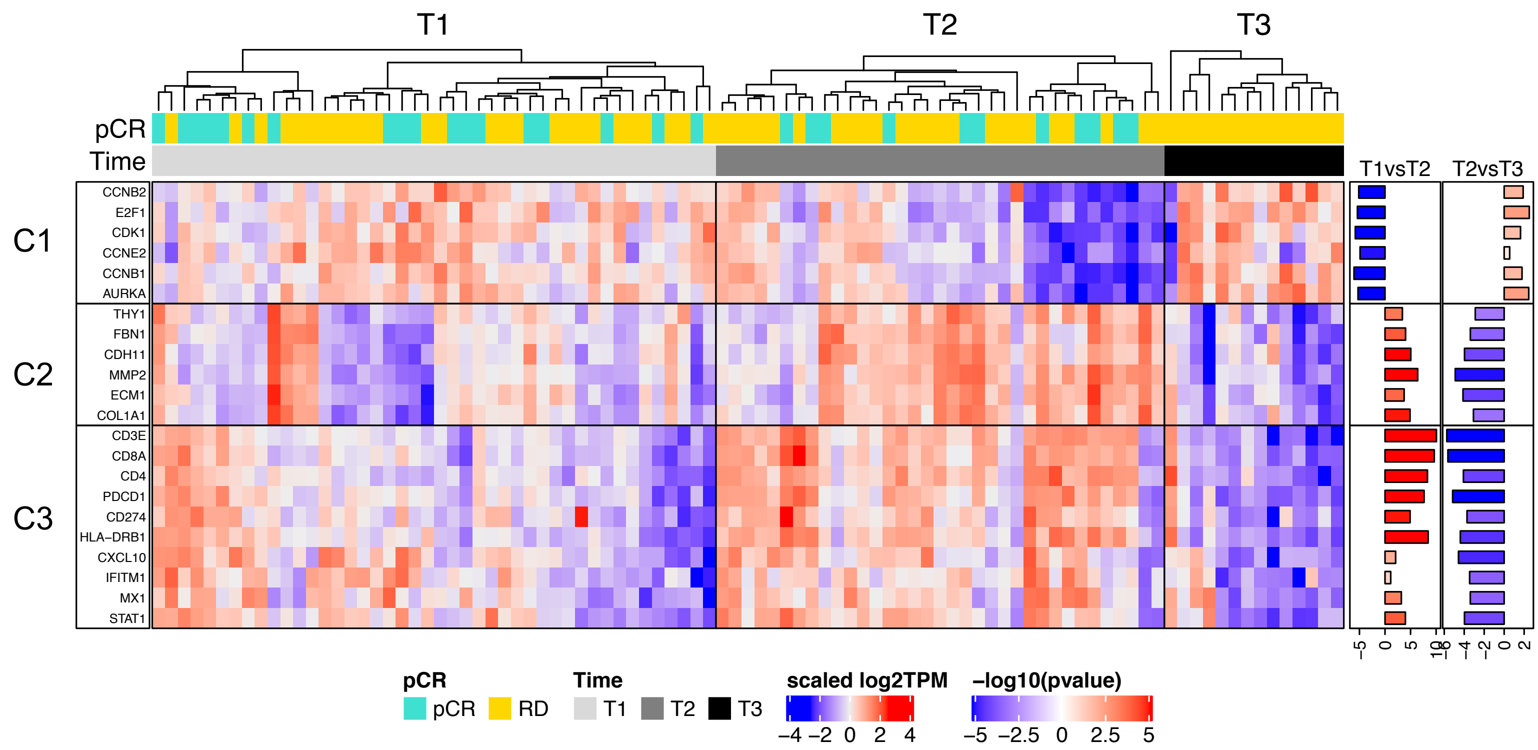

Expression patterns of representative genes from cell cycle, EMT and immune related pathways in ER+ (a), ER+ combined (b), HER2+ (c), HER2+ combined (d) and TN (e) subtypes. Gene expression in log2(TPM) was centered and scaled to z-scores for each gene. Statistical significance of DE was determined using LMER and shown by the bar plots on the right as sign*-log10(p-value) based on the sign of the t-statistics. Source data are provided as a Source Data file.

Supplementary Figure 3. Comparisons of Subtype DE patterns

a

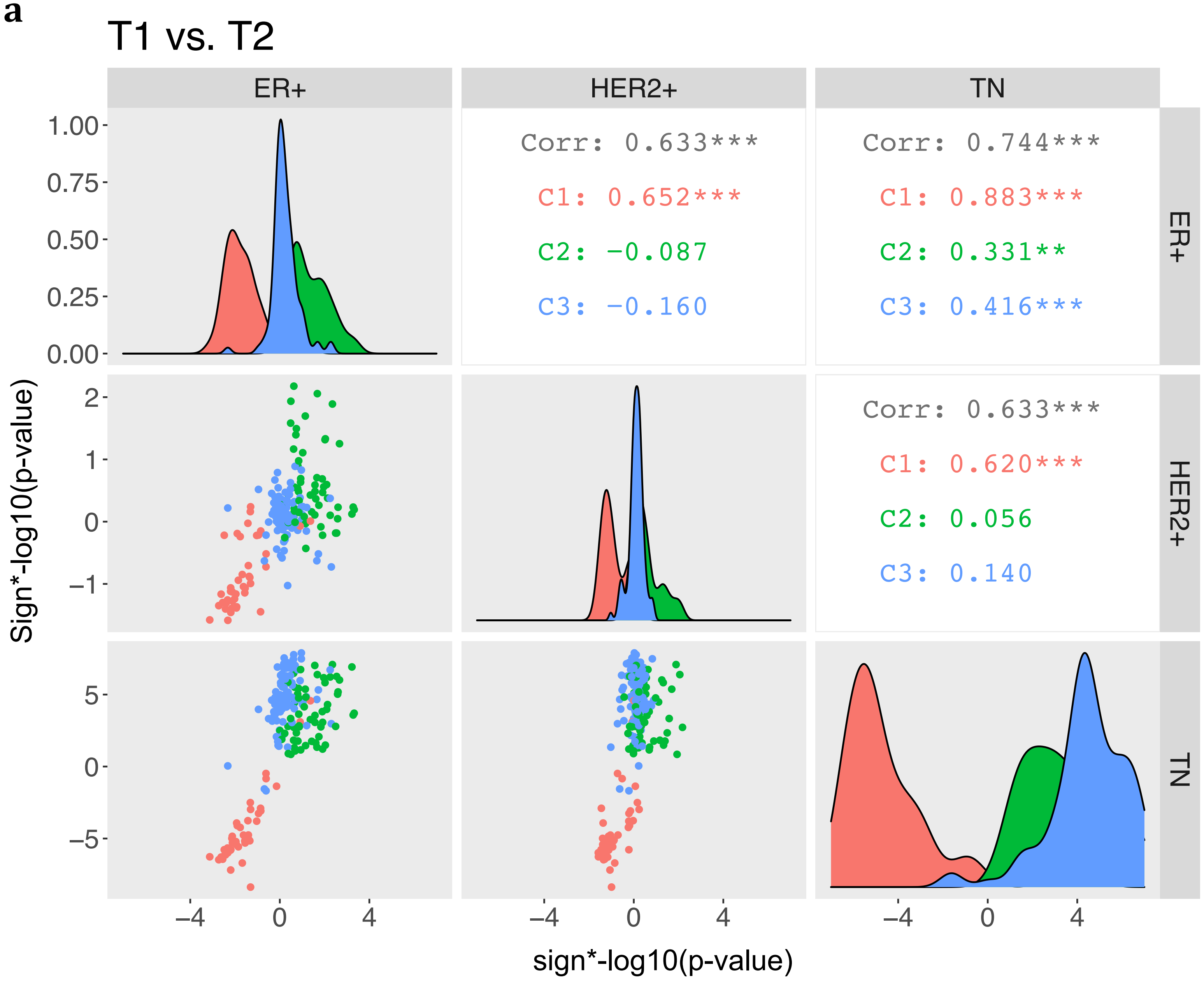

Supplementary Figure 3. Comparisons of Subtype DE patterns

b

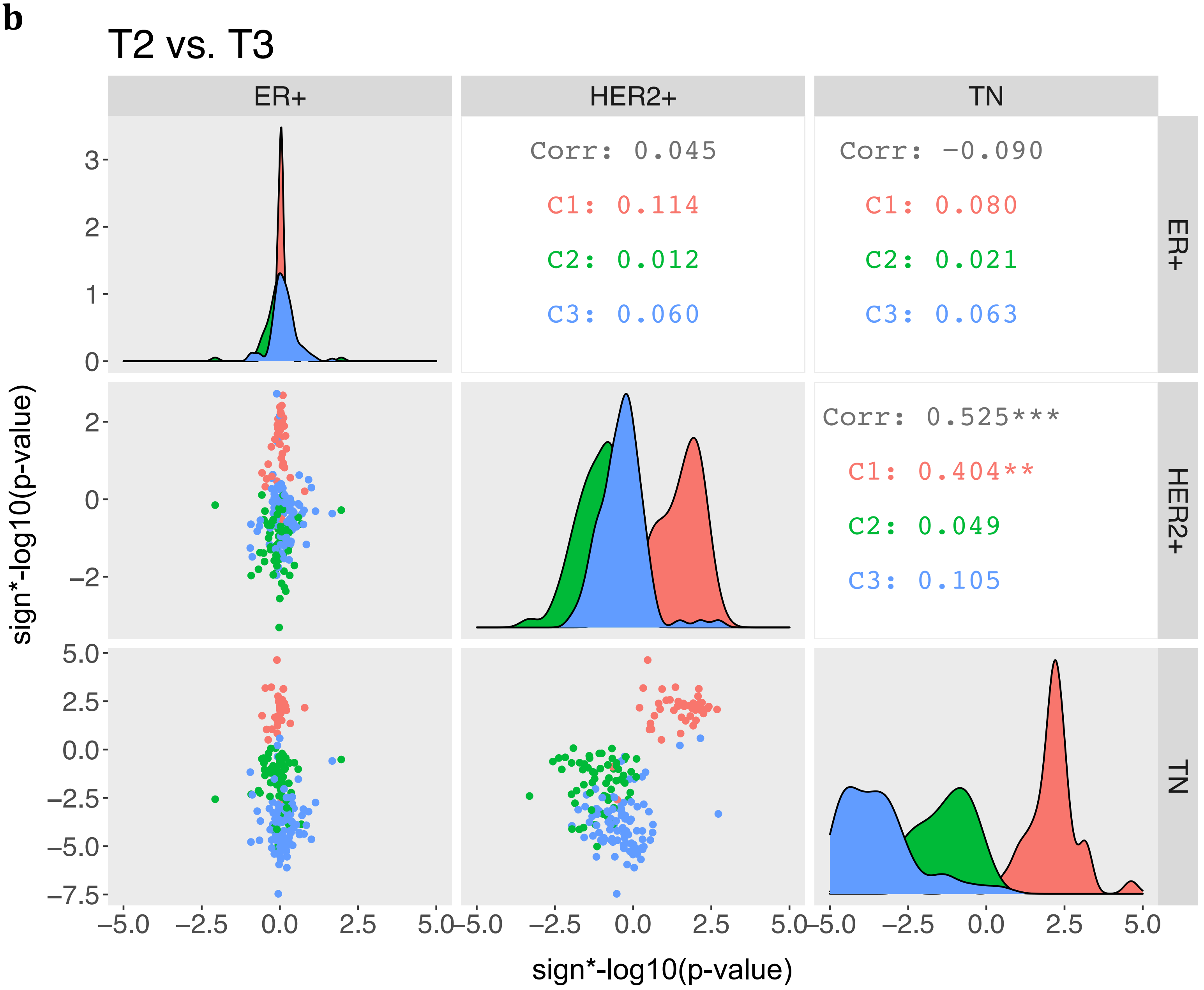

Supplementary Figure 3. Comparisons of Subtype DE patterns

c

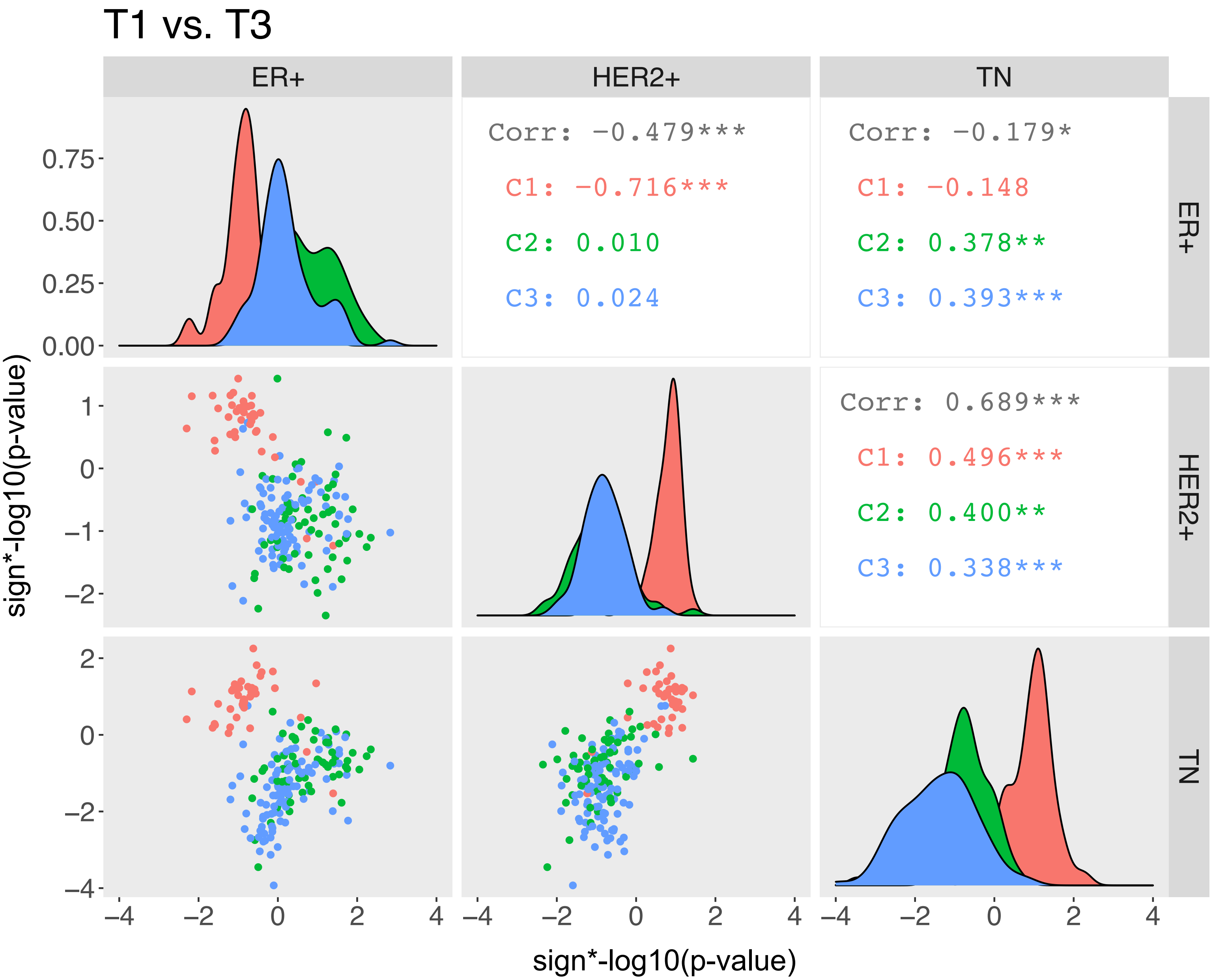

Supplementary Figure 3. Comparisons of Subtype DE patterns

d

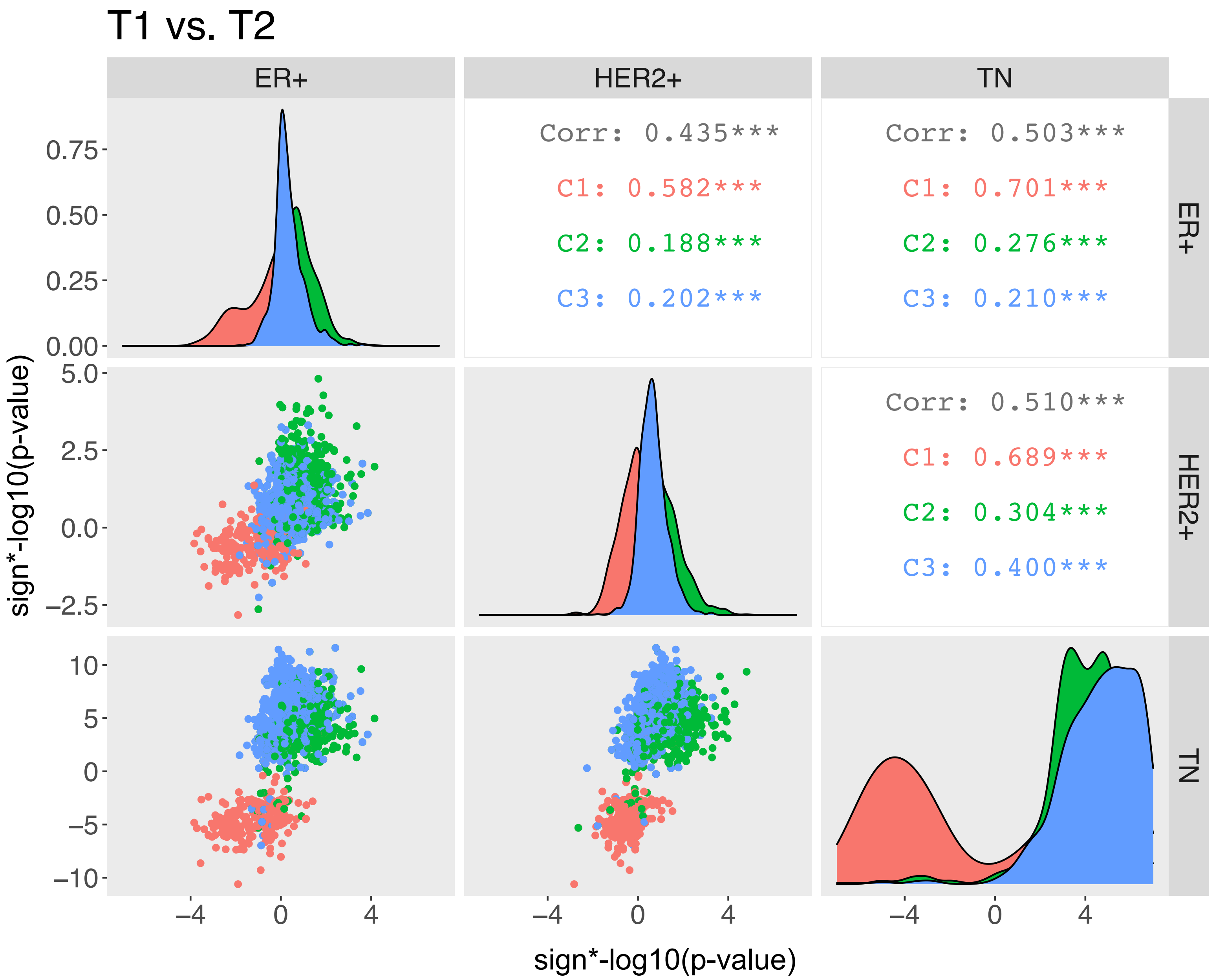

Supplementary Figure 3. Comparisons of Subtype DE patterns

e

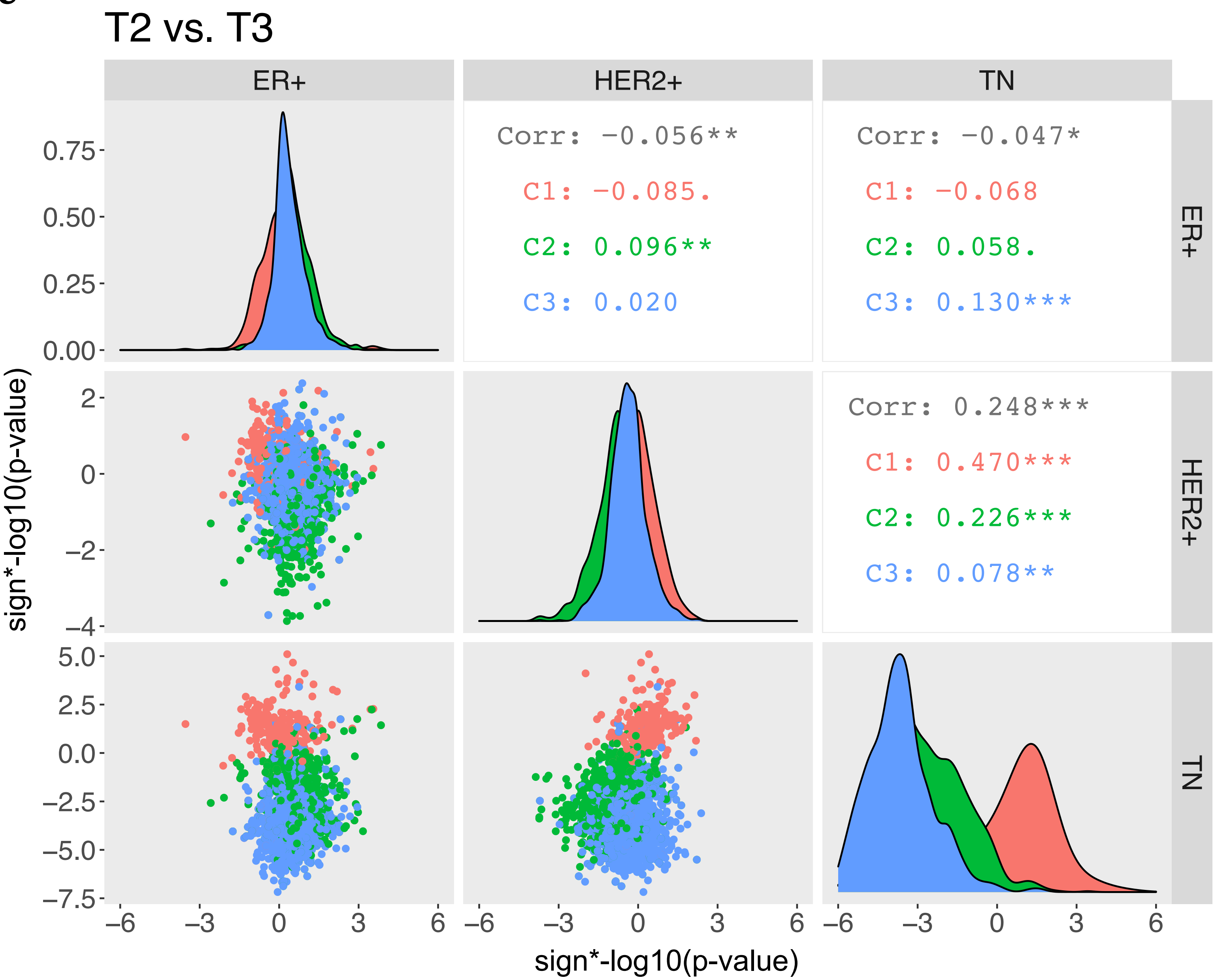

Supplementary Figure 3. Comparisons of Subtype DE patterns

f

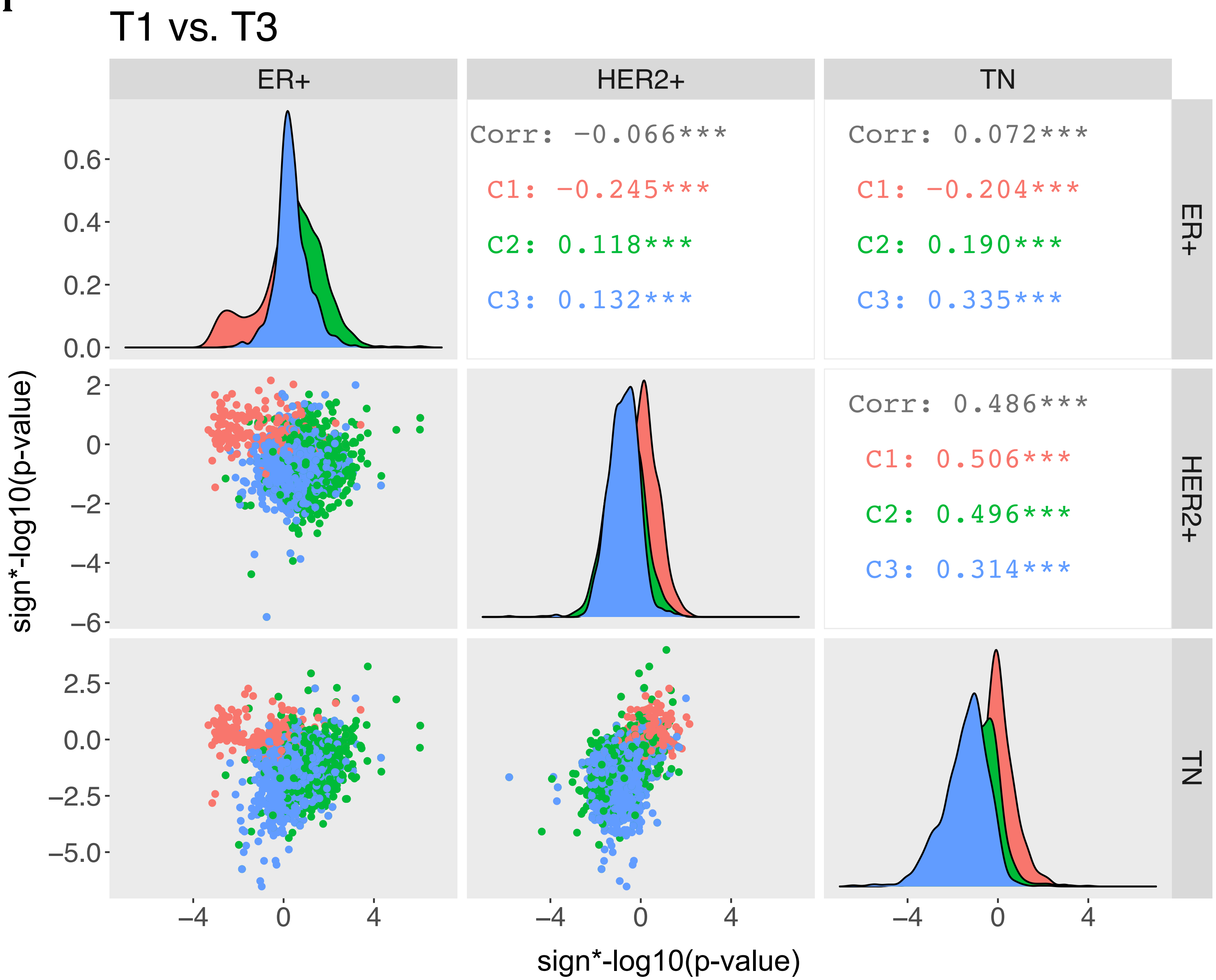

Spearman correlations of the DE statistical significance for pathways (a-c) and genes (d-f), grouped by DE cluster mapping, among ER+, HER2+ and TN subtypes in T1 vs. T2 (a, d), T2 vs. T3 (b, e) and T1 vs. T3 (c, f) comparisons. Asterisks indicate statistical significance of the correlation: '*' 0.01<*p*<0.05; '**' 0.001<p<0.01. '***' p<0.001. The statistical significance of DE was determined by LMER and represented by sign*-log10(p-value) based on the sign of the t-statistics. Source data are provided as a Source Data file.

Supplementary Figure 4: IO Predictive immune signature is enriched on-treatment in TNBC RD cases.

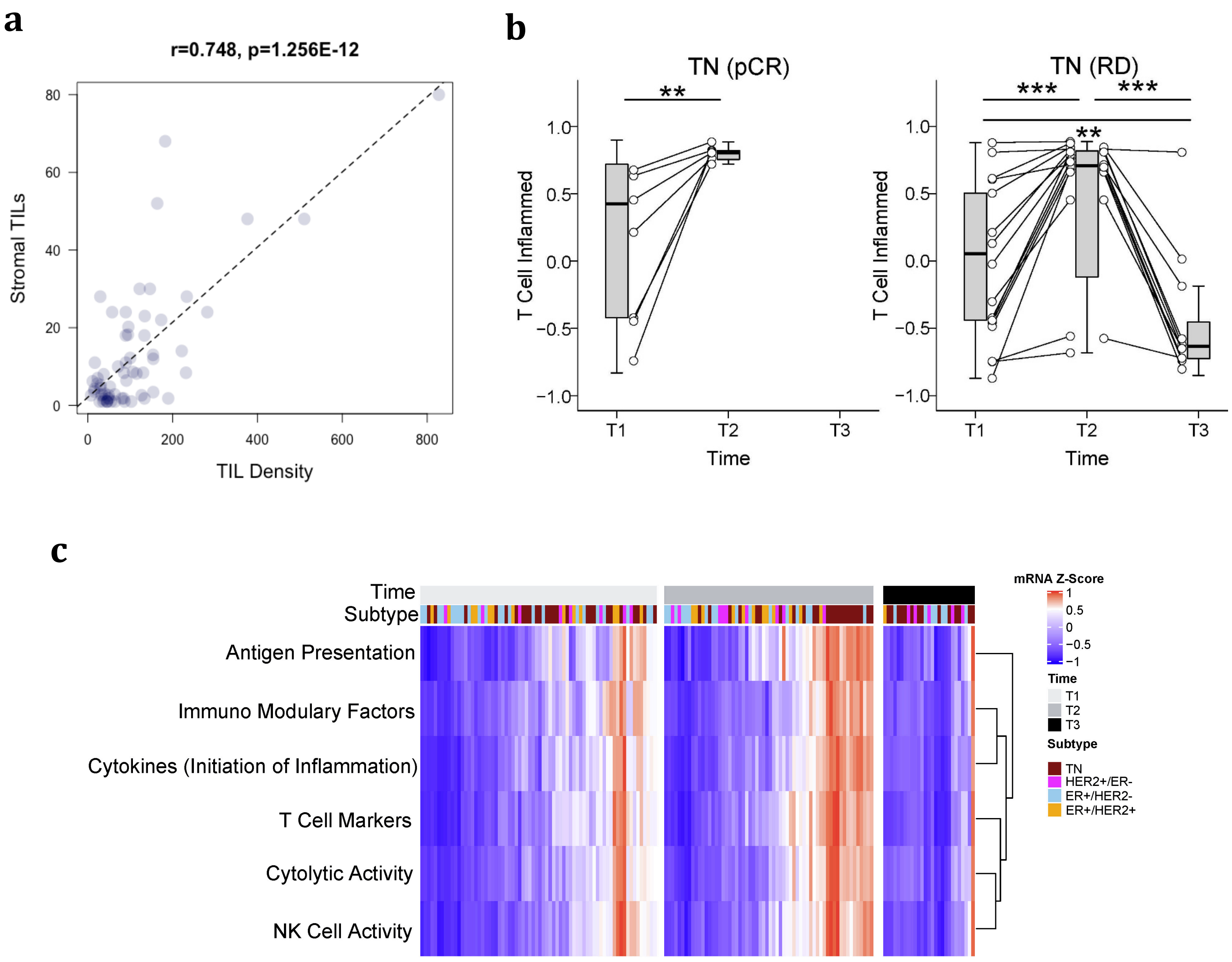

(a) Scatter plot showing Spearman correlation of stromal TIL score vs. TIL density (r = 0.748, p=1.256E-12). (b) Changes in the T cell inflamed signature over time and stratified by pCR status in TN samples. The box is bounded by the first and third quartile with a horizontal line at the median and whiskers extend to the maximum and minimum value. Asterisks indicate statistical significance based on LMER: ** $0.001<p<0.01$; *** $p<0.001$. See Supplementary table 2 for exact p-values. (c) Expression pattern of the 6 components of the expanded immune signature in all samples from the RD cohort. Source data are provided as a Source Data file.

Supplementary Figure 5: NAC induced changes in TIL subsets differ by functional roles

a

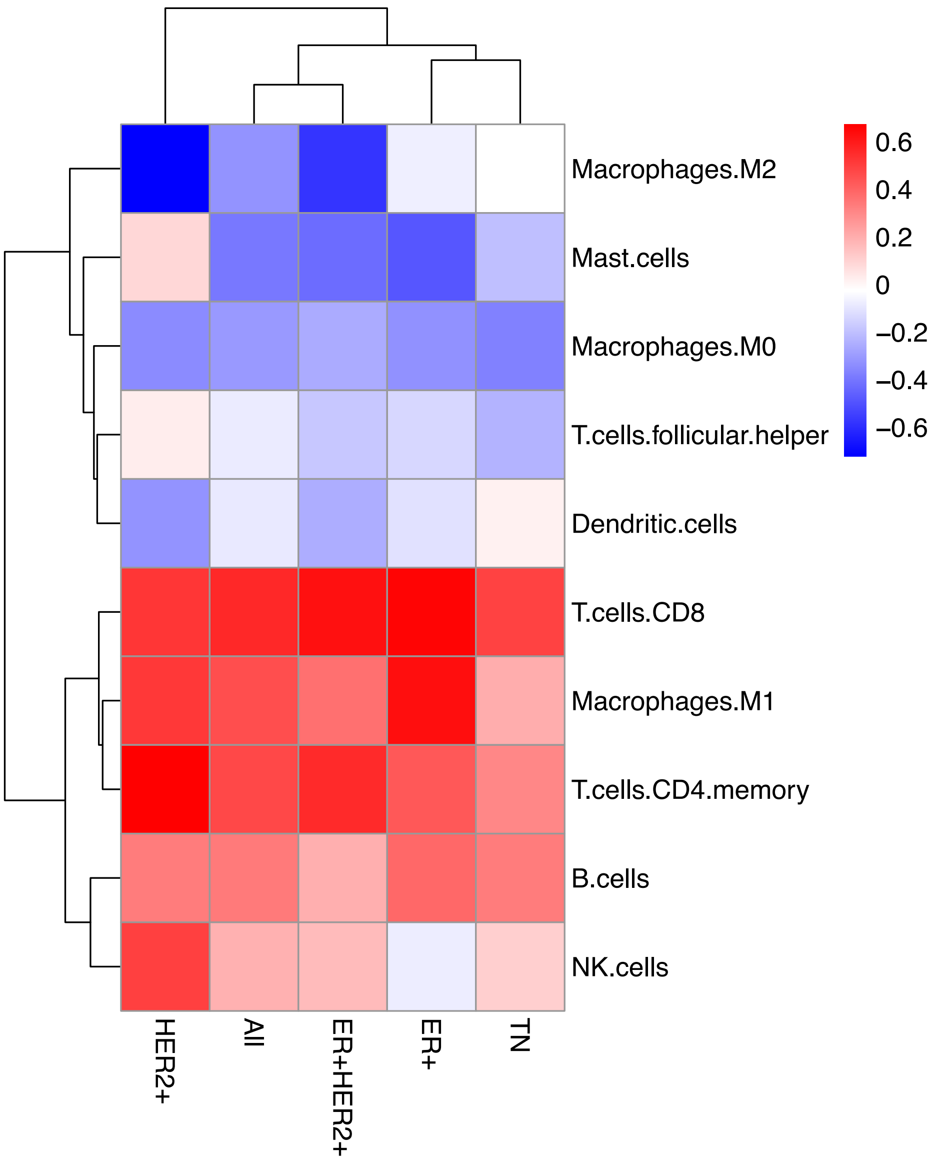

Supplementary Figure 5: NAC induced changes in TIL subsets differ by functional roles

b

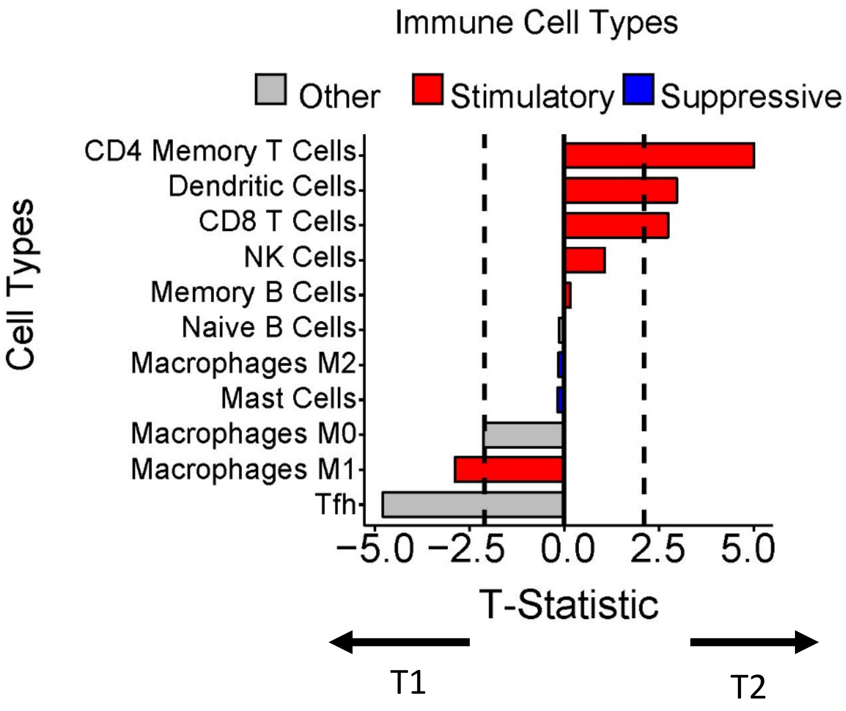

c

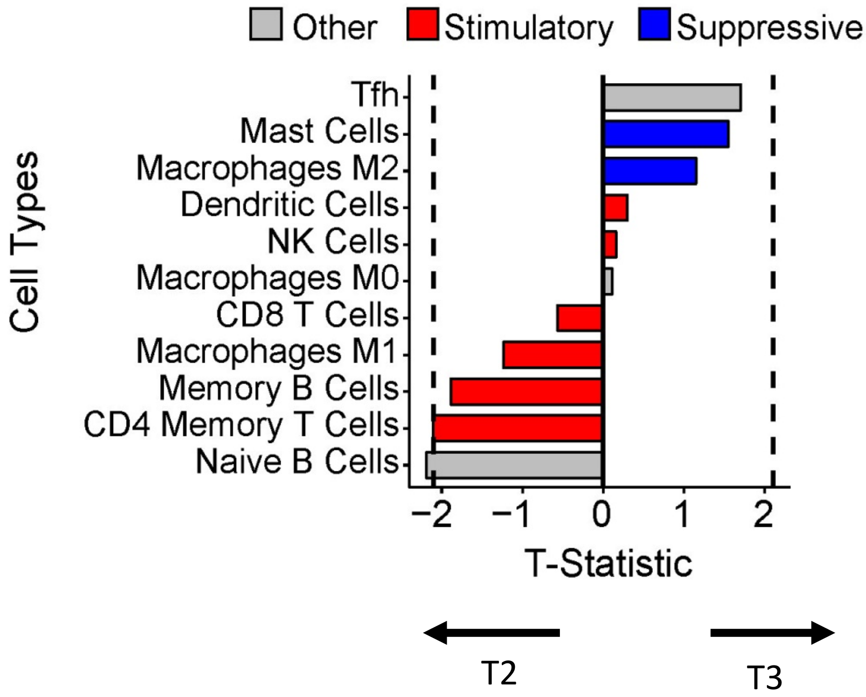

(a) Spearman correlations between immune cell fractions vs. CYT score overall and in different subtypes. (b-c) Waterfall plots showing the change in estimated immune cell fractions over time. X-axis shows t-statistics from multiple mixed effects regression. Dotted lines indicate statistical significance ($p<0.05$). Source data are provided as a Source Data file.

Supplementary Figure 6: NAC induced dynamic changes in tumor associated immune states

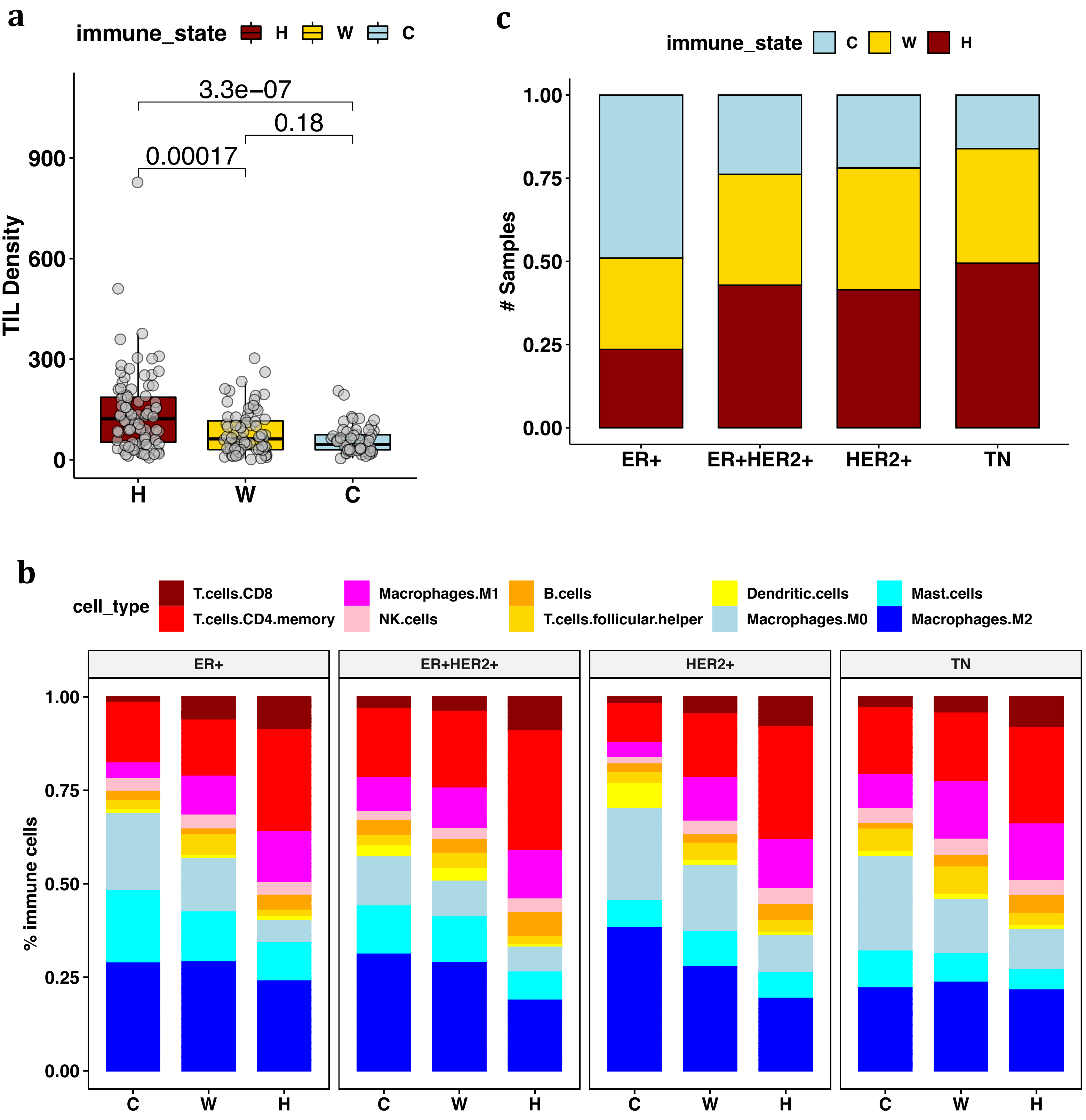

Supplementary Figure 6: NAC induced dynamic changes in tumor associated immune states

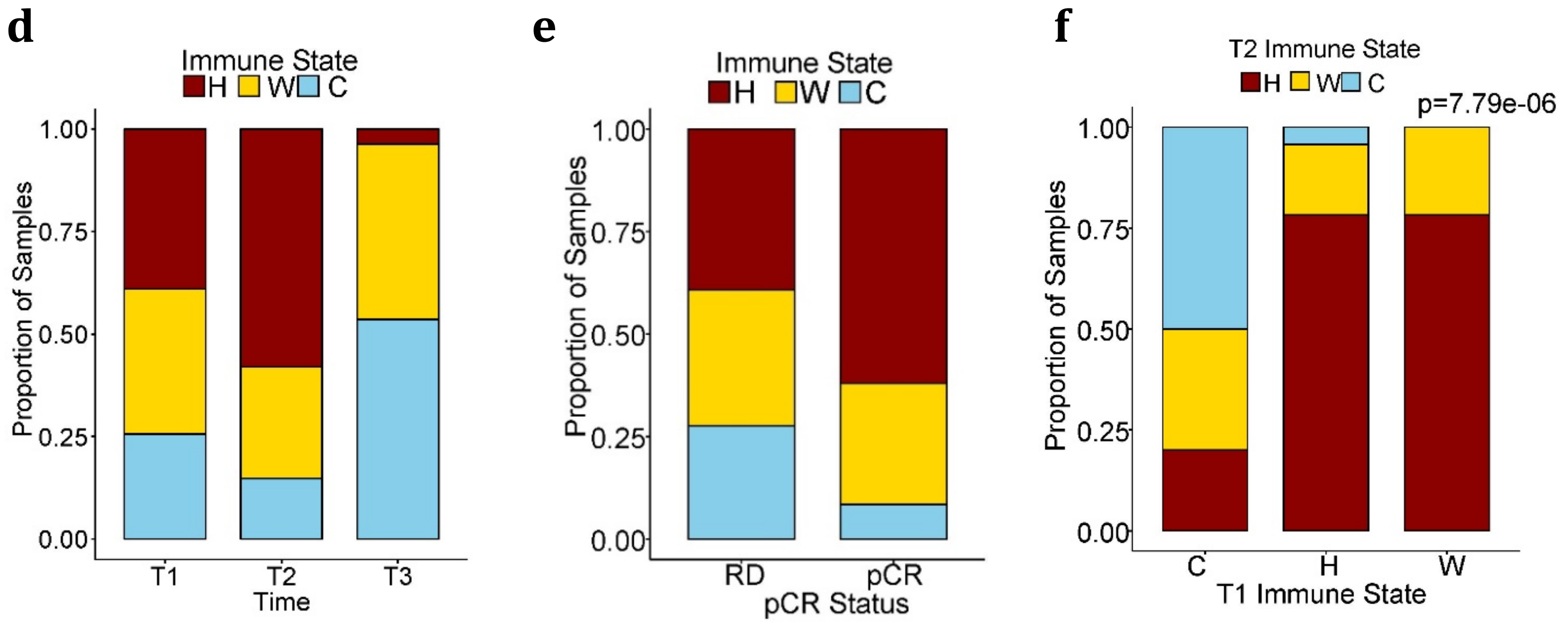

(a) Distributions of TIL density across three immune states. The box is bounded by the first and third quartile with a horizontal line at the median and whiskers extend to the maximum and minimum value. Statistical significance was determined using two-sided Wilcoxon rank sum test with the following sample sizes – C: n=59; W=75; H=93. (b) Distributions of immune cell fractions across subtypes and immune states. (C-E) Distributions of immune states across subtypes (c), treatment times (d) and pCR statuses for T1 and T2 samples (e). (f) Distributions of T2 immune states vs. T1 immune states. Source data are provided as a Source Data file.

Supplementary Figure 7: Distribution patterns of tumor purity

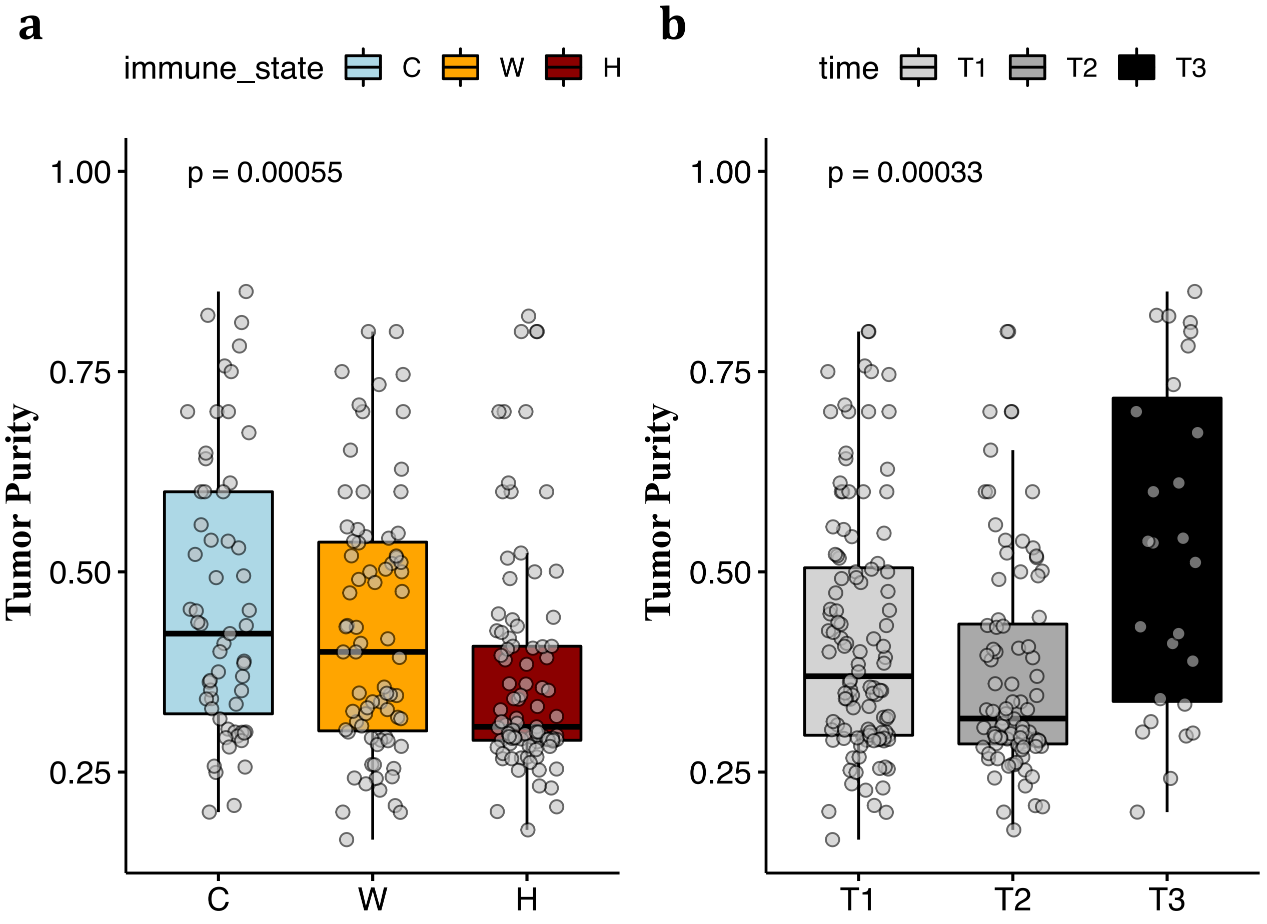

Distributions of tumor purity over the immune states (a) and treatment times (b). C: Cold,; W: Warm; H: Hot. T1: baseline; T2: on-treatment; T3: surgery. The box is bounded by the first and third quartile with a horizontal line at the median and whiskers extend to the maximum and minimum value. Statistical significance was determined using two-sided Kruskal-Wallis test with the following sample sizes – C: n=59; W=75; H=93; T1: n=112; T2: n=88; T3: n=27. Source data are provided as a Source Data file.

Supplementary Figure 8: Correlation analysis of TME factors

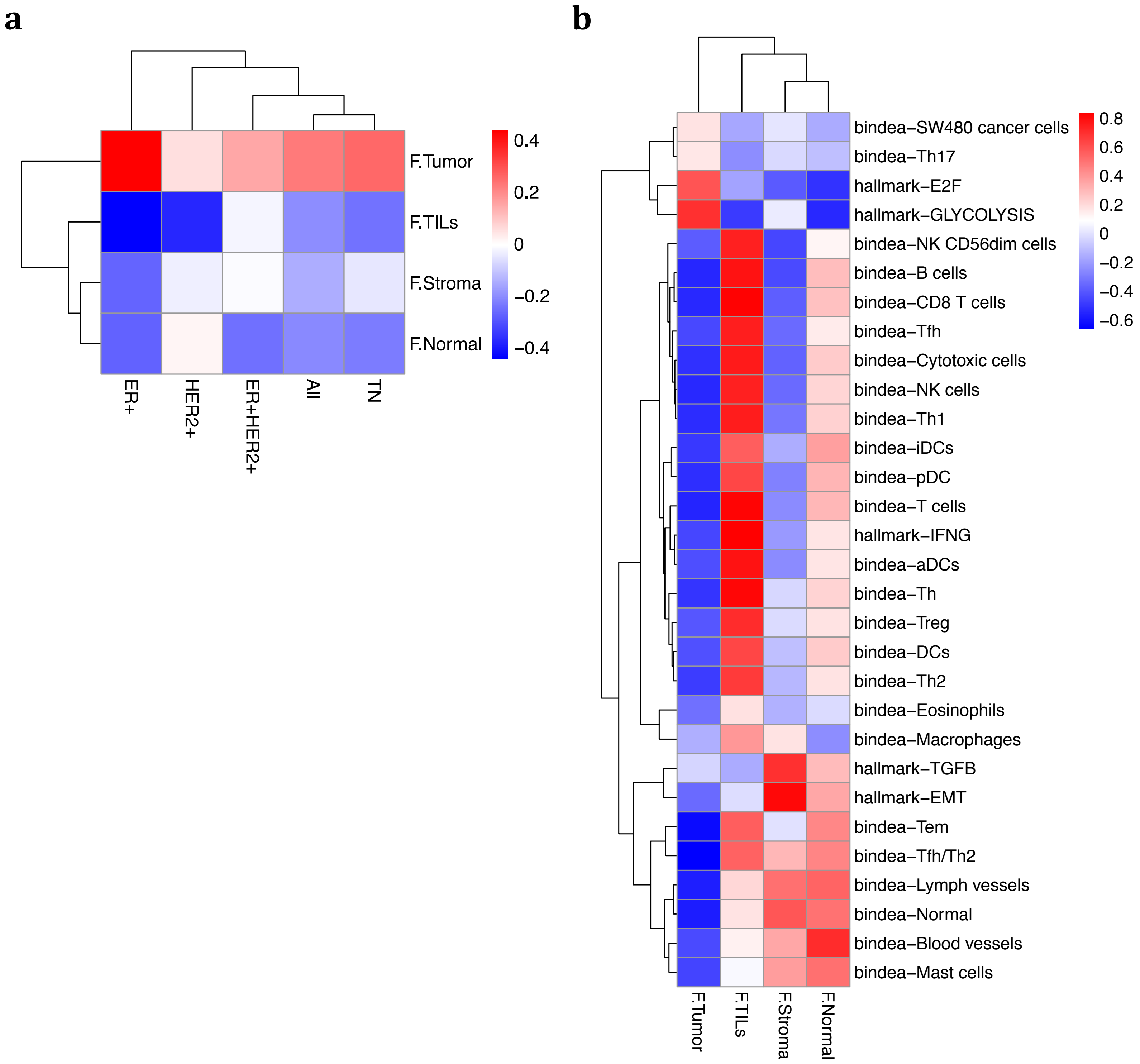

Supplementary Figure 8: Correlation analysis of TME factors

c

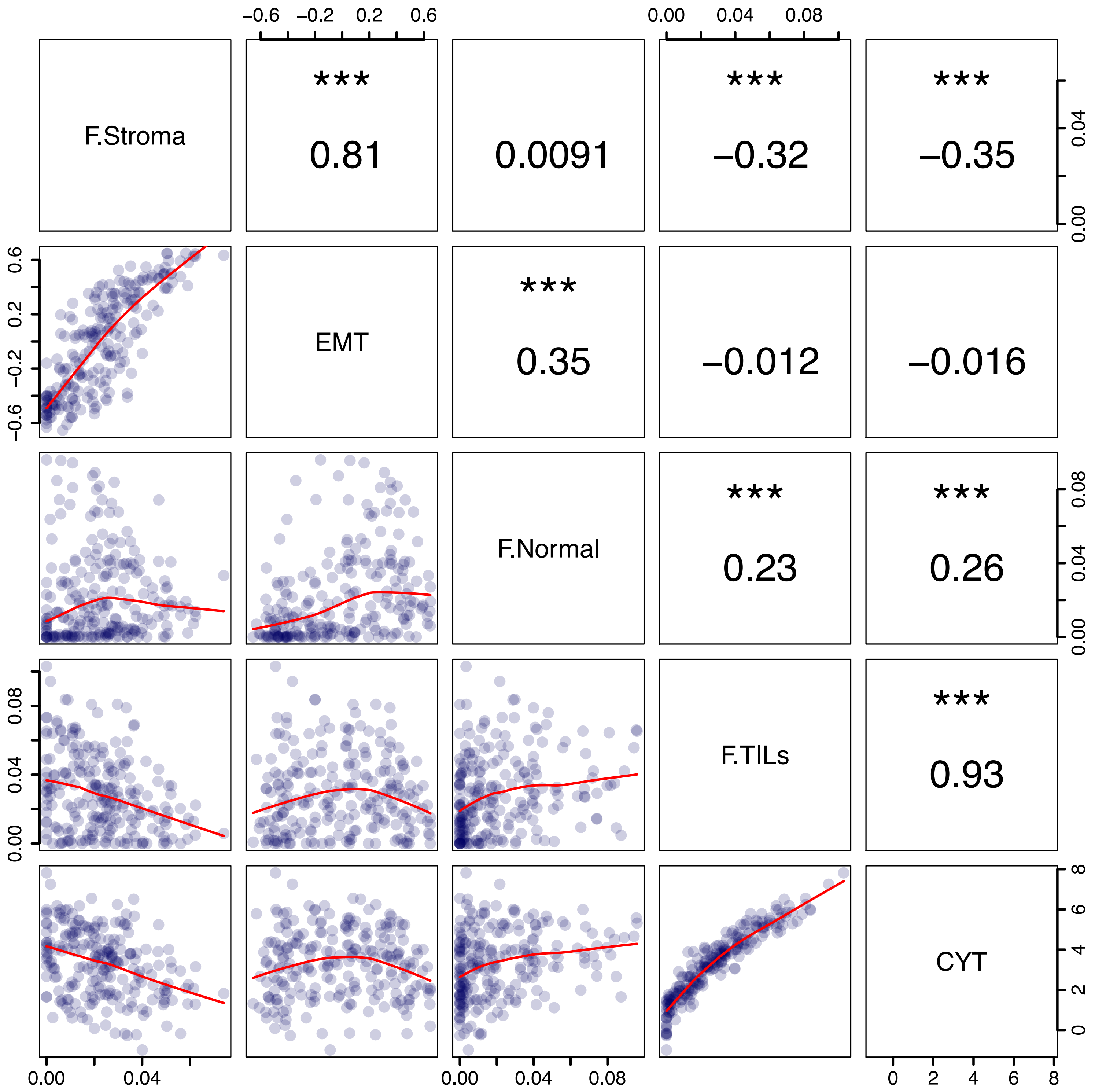

(a) Correlations between tumor purity and NMF factors in different sample groups. (b) Correlations of NMF factors (columns) vs. expression signatures (rows). Shown in the heatmaps are Pearson correlation coefficients. Source data are provided as a Source Data file. (c) Pairwise correlations of three TME factors vs. Hallmark EMT signature and log2(CYT) scores. Scatter plots and lowess fit were shown in lower panels. Pearson correlation coefficients are shown in the upper panels. *** $p < 0.001$.

Supplementary Figure 9: Distribution patterns of TME factors

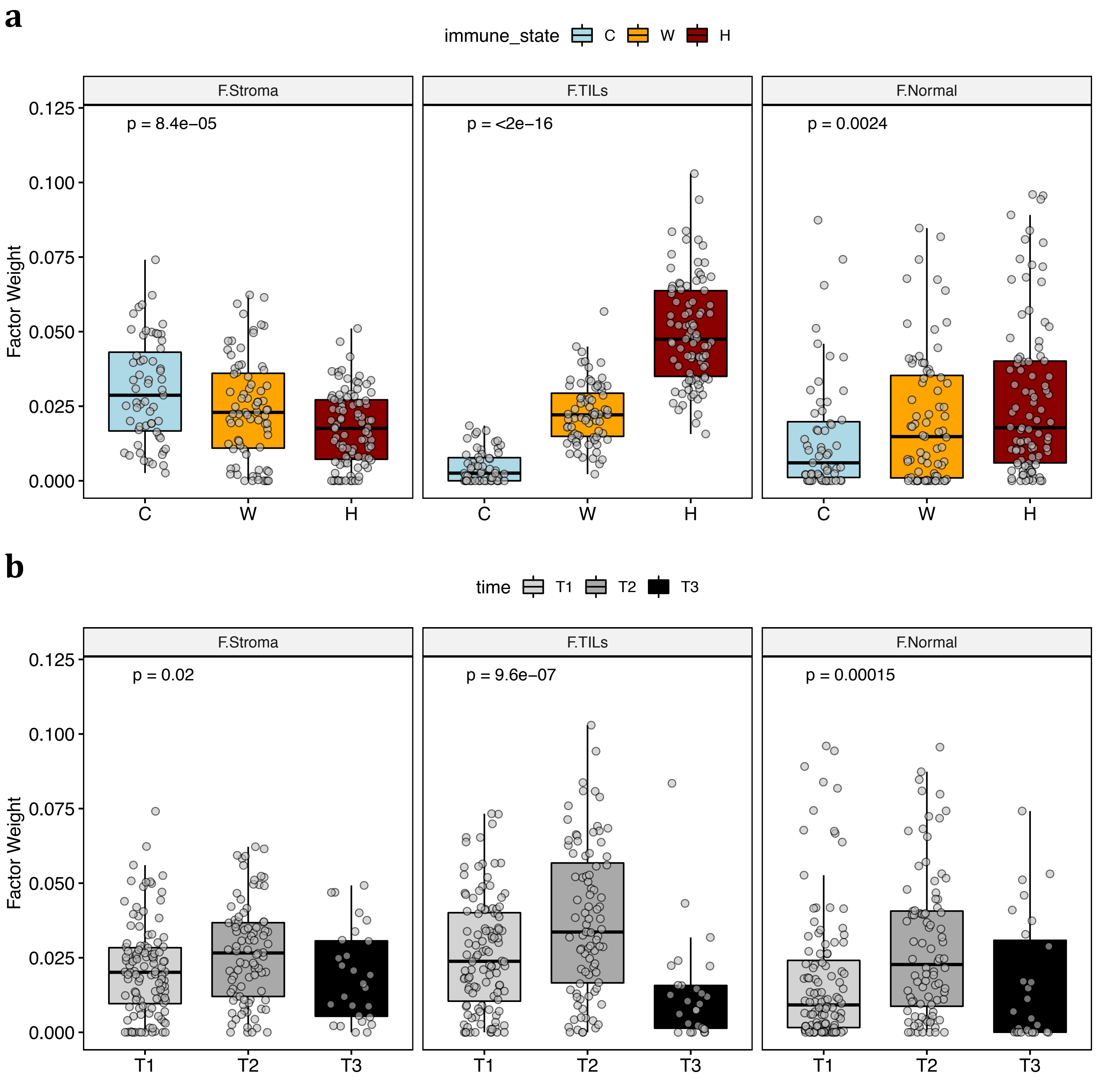

Distribution of three TME factors – F-TILs, F-Normal and F-Stroma – over immune states (a) and treatment times (b). The box is bounded by the first and third quartile with a horizontal line at the median and whiskers extend to the maximum and minimum value. Statistical significance was determined using two-sided Kruskal-Wallis test with the following sample sizes – C: n=59; W=75; H=93; T1: n=112; T2: n=88; T3: n=27. Source data are provided as a Source Data file.

Supplementary Figure 10: F-TILs is the only TME factor associated with clinical response

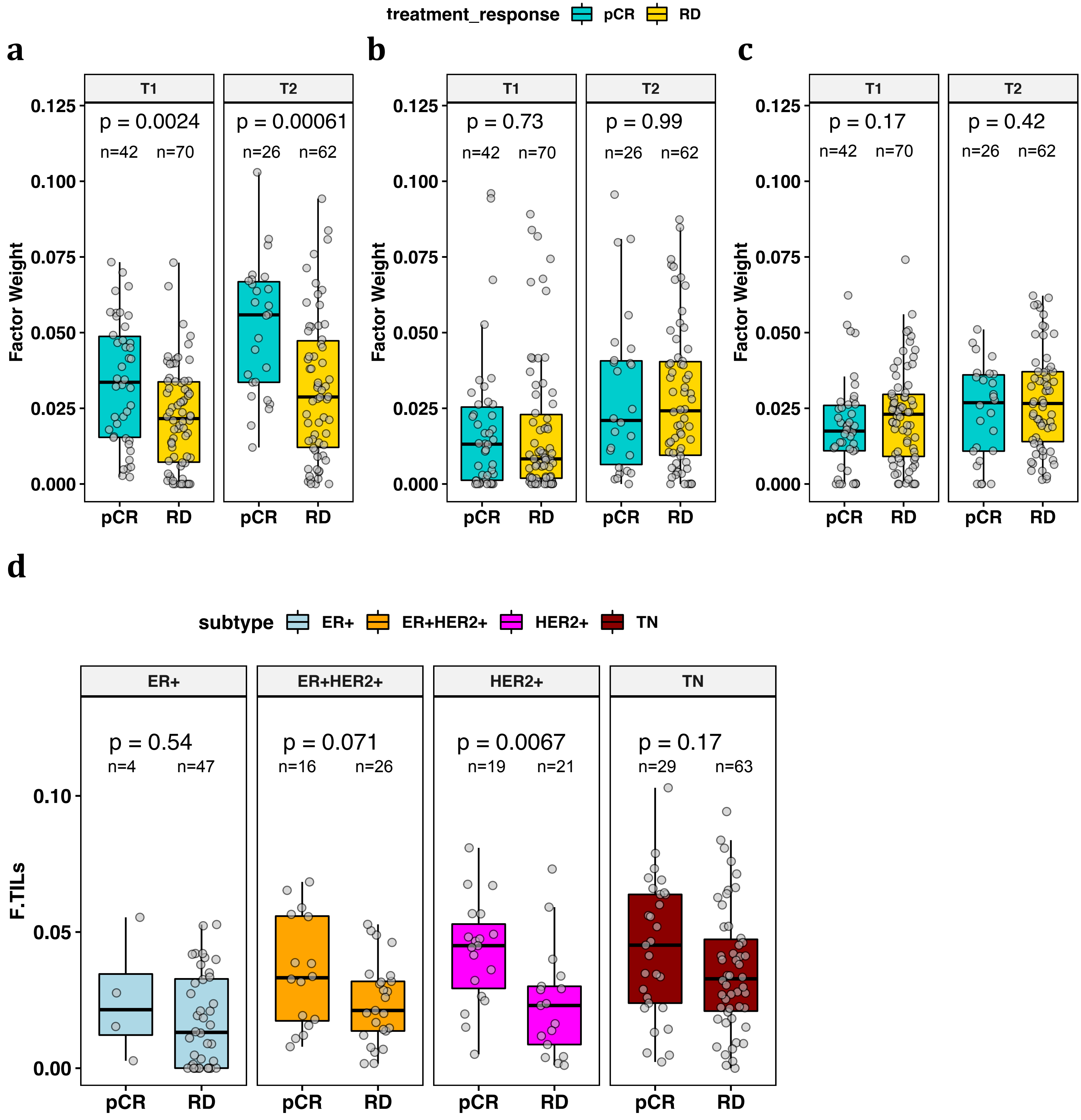

(a-c) Distribution of three TME factors – F-TILs (a), F-Normal (b) and F-Stroma (c) vs. pCR status. (d) Distribution of F-TILs vs. pCR status across subtypes. The box is bounded by the first and third quartile with a horizontal line at the median and whiskers extend to the maximum and minimum value. Statistical significance was determined using two-sided Wilcoxon test. Source data are provided as a Source Data file.

Supplementary Figure 11: TIL abundance associated with clinical response

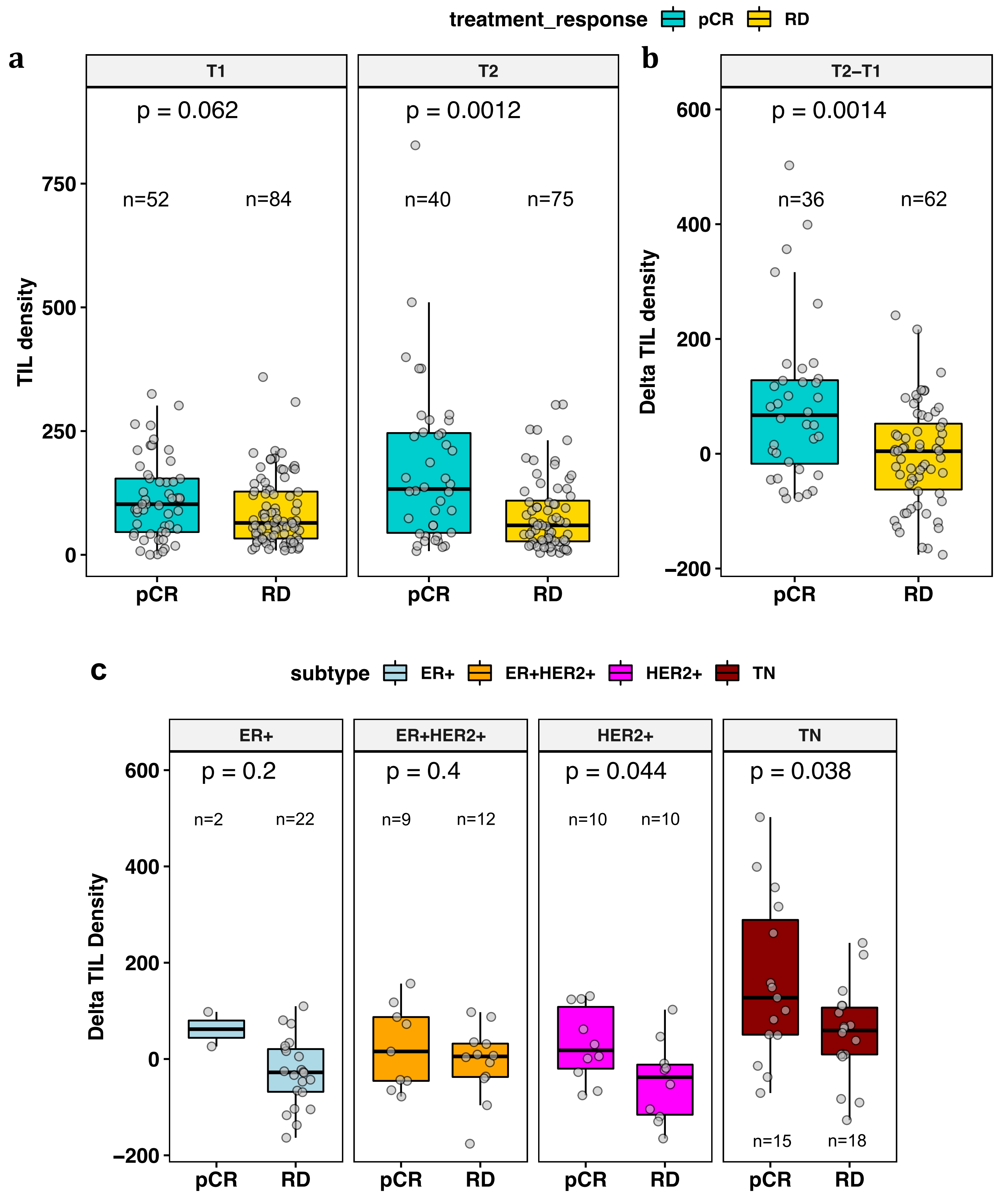

Supplementary Figure 11: TIL abundance associated with clinical response

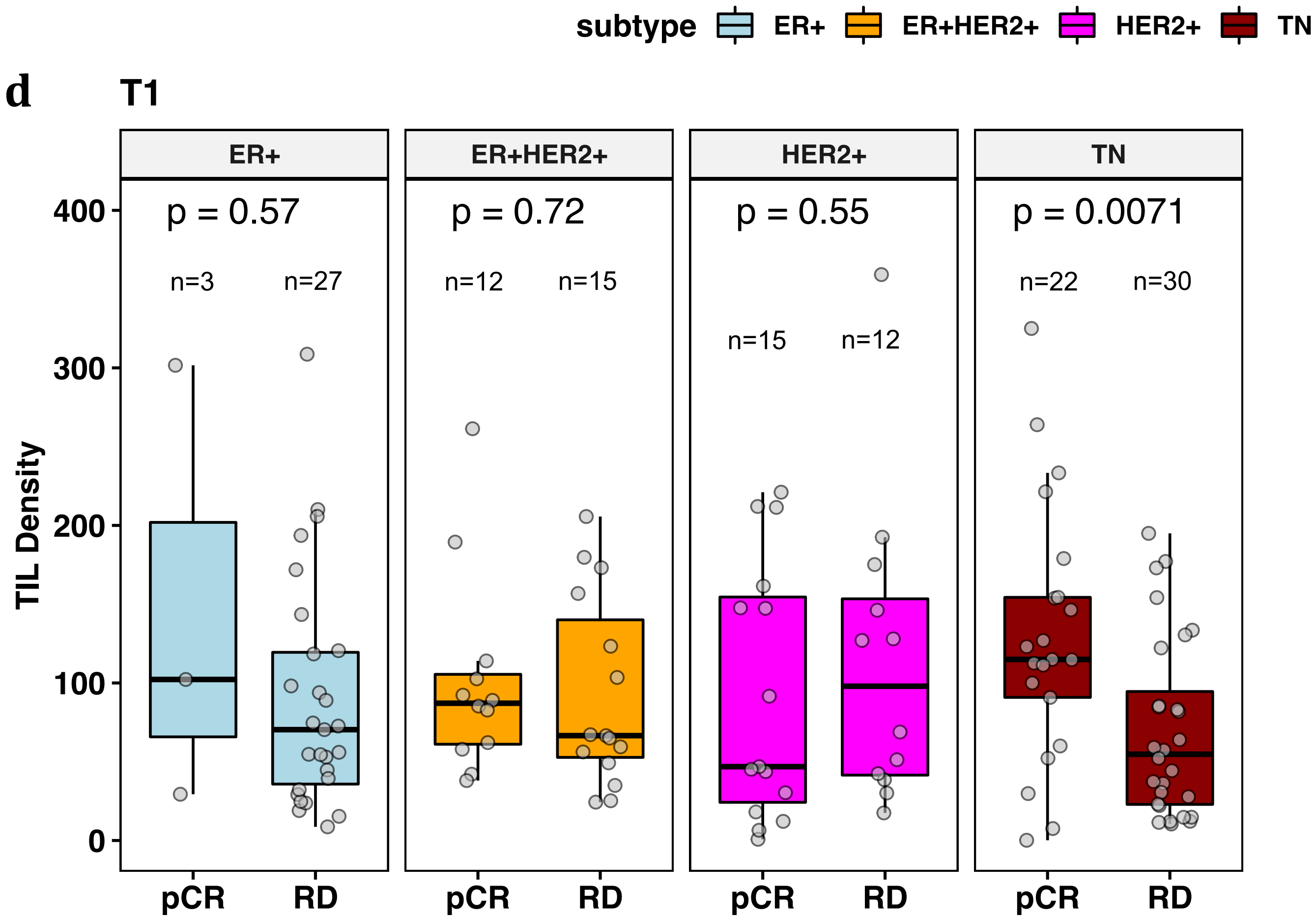

Supplementary Figure 11: TIL abundance associated with clinical response

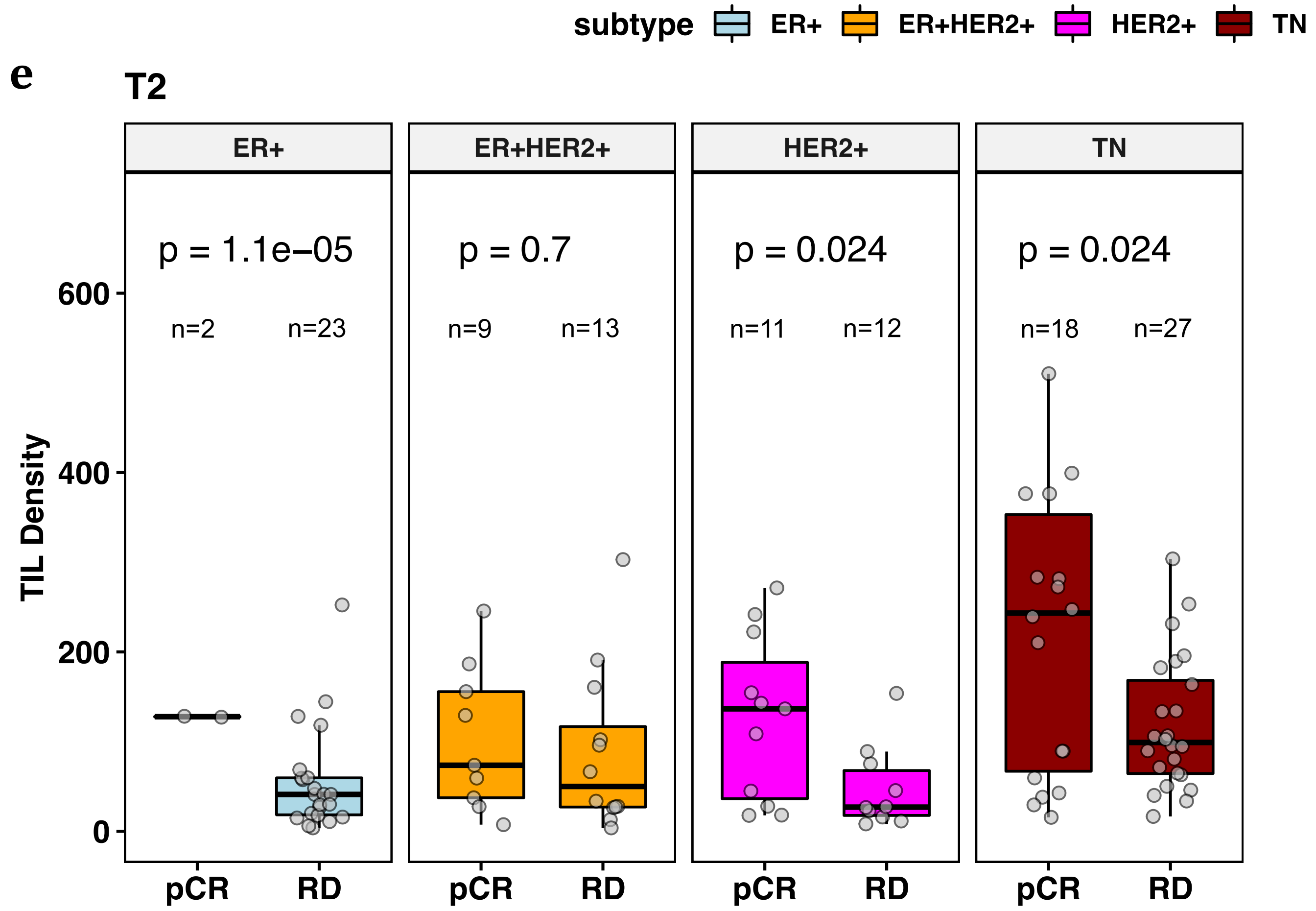

(a) TIL density distribution vs. pCR status over the entire cohort at T1 and T2. (b) Distribution of delta TIL density, difference in TIL density between T1 and T2 vs. pCR status. (c) Distributions of delta TIL density vs. pCR status and subtypes. For all box plots, the box is bounded by the first and third quartile with a horizontal line at the median and whiskers extend to the maximum and minimum value. Statistical significance was determined using two-sided Wilcoxon test. (d-e) Comparisons of TIL density across pCR status and subtypes at T1 (d) and T2 (e). Source data are provided as a Source Data file.

Supplementary Figure 12: Association of immune cell types vs. pCR status

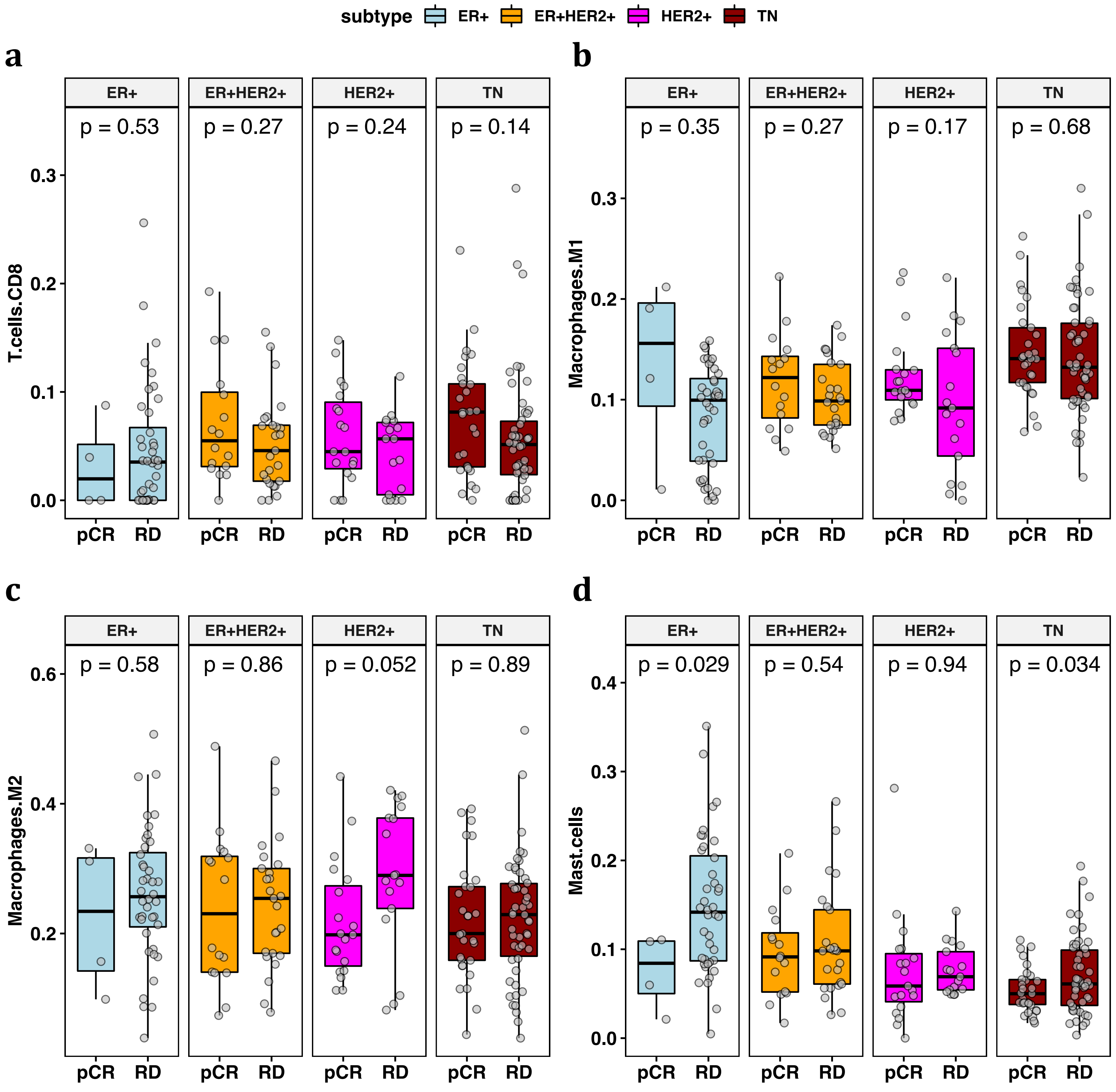

Supplementary Figure 12: Association of immune cell types vs. pCR status

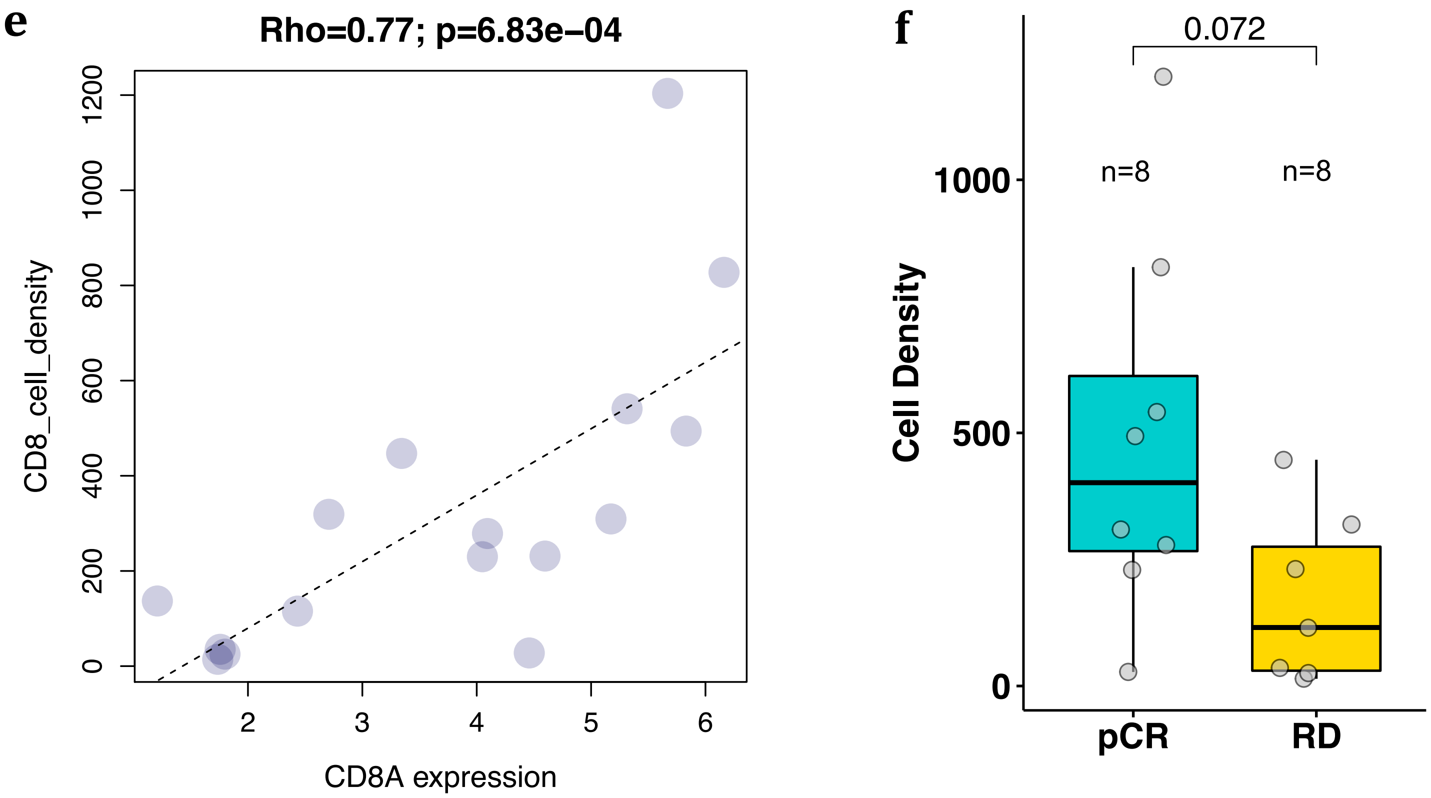

(a-d) Distribution of relative immune cell fractions vs. pCR status across subtypes for CD8+ T cells (a), M1 Macrophage (b), M2 Macrophage (c) and Mast cells (d). The box is bounded by the first and third quartile with a horizontal line at the median and whiskers extend to the maximum and minimum value. Statistical significance was determined using two-sided Wilcoxon test with the following sample sizes – ER+: n=4 (pCR) and n=47 (RD); ER+/HER2+: n=16 (pCR), n=26 (RD); HER2+: n=21 (pCR), n=22 (RD); TN: n=31 (pCR), n=65 (RD). Source data are provided as a Source Data file. (e) Spearman correlation of CD8+ cell density vs. CD8A gene expression. (f) Distribution of CD8+ cell density vs. pCR status. The box is bounded by the first and third quartile with a horizontal line at the median and whiskers extend to the maximum and minimum value. Statistical significance was determined using two-sided Wilcoxon test.

Supplementary Table 1: Clinical data summary

Clinical attribute	RD	pCR	# cases
**Subtype (diagnosis)**	(n = 84)	(n = 52)	(n = 136)
ER+/HER2-	27 (32%)	3 (6%)	30 (22%)
ER+/HER2+	15 (18%)	12 (23%)	27 (20%)
HER2+/ER-	12 (14%)	15 (29%)	27 (20%)
TN	30 (36%)	22 (42%)	52 (38%)
**Menopausal Status**	(n = 84)	(n = 52)	(n = 146)
Premenopausal	71 (78%)	33 (60%)	104 (71%)
Postmenopausal	20 (22%)	20 (36%)	40 (27%)
N/A	0 (0%)	2 (4%)	2 (2%)
**Neoadjuvant Treatment**	(n = 84)	(n = 52)	(n = 146)
AC+T	59 (65%)	23 (42%)	82 (56%)
AC+T+Trastuzumab	24 (26%)	23 (42%)	47 (32%)
Others	8 (9%)	9 (16%)	17 (11%)
**Ki67**	(n = 84)	(n = 52)	(n = 146)
0-25%	20 (22%)	8 (15%)	28 (19%)
25-50%	30 (33%)	23 (42%)	53 (36%)
50-75%	18 (20%)	4 (7%)	22 (15%)
75≤%	22 (24%)	20 (36%)	42 (29%)
N/A	1 (1%)	0 (0%)	1 (1%)
**Clinical Tumor Stage (diagnosis)**	(n = 84)	(n = 52)	(n = 146)
T1	5 (6%)	6 (11%)	11 (8%)
T2	46(50%)	33 (60%)	79 (54%)
T3	21 (23%)	14 (25%)	35 (24%)
T4	14 (15%)	1 (2%)	15 (10%)
N/A	5 (6%)	1 (2%)	6 (4%)
**Clinical Nodal Stage (diagnosis)**	(n = 84)	(n = 52)	(n = 146)
N0	3 (3%)	1 (2%)	4 (3%)
N1	26 (29%)	22 (40%)	48 (33%)
N2	30 (33%)	23 (41%)	53 (36%)
N3	27 (30%)	8 (15%)	35 (24%)
N/A	5 (5%)	1 (2%)	6 (4%)
**Histological Type**	(n = 84)	(n = 52)	(n = 146)
Invasive ductal carcinoma	86 (95%)	54 (98%)	140 (96%)
Invasive carcinoma with lobular features	3 (3%)	0 (0%)	3 (2%)
Other	2 (2%)	1 (2%)	3 (2%)
**Surgery Type**	(n = 84)	(n = 52)	(n = 146)
Partial Mastectomy	43 (47%)	42 (76%)	85 (58%)
Modified Radical Mastectomy	48 (53%)	13 (24%)	61 (42%)

A table of summary statistics showing the numbers (n) and percentages of patients in different categories as grouped by key clinical attributes (row) and treatment outcome (column). RD: residual disease. pCR: pathologic complete response. N/A: not available.

Supplementary Table 2: Associations of TILs vs. treatment time

Feature	Subtype	P-value (pCR, T1-T2)	P-value (RD, T1-T2)	P-value (RD, T2-T3)	P-value (RD, T1-T3)
TIL Density	ALL	0.01277856	0.359847	0.07299099	0.03137544
TIL Density	TN	0.03950141	0.007006734	0.01689376	5.491065E-01
TIL Density	Non-TN	0.05188501	0.003318955	0.2536138	0.000287992
TIL Density	HER2+/ER-	0.4656703	0.07296769	0.4594605	0.02573998
TIL Density	ER+/HER2+	0.07411095	0.2057332	NA	NA
TIL Density	ER+/HER2-	1	0.03552127	0.539699	0.01341056
Stromal TIL	TN	8.33E-05	4.90E-03	5.81E-03	4.40E-03
IFNG	TN	0.02712183	2.32E-06	1.93E-07	0.0133623
T Cell Inflammed	TN	0.006652708	6.37E-07	1.65E-07	0.007640375
Preliminary Expanded Immune	TN	0.008488697	3.78E-07	8.64E-08	0.009167474

Estimates of the statistical association between immune features and treatment times in different subsets of the cohort as grouped by breast cancer subtype (row) and treatment outcome (column). Statistical significance was calculated using linear mixed effects regression (LMER) adjusting for tumor purity as covariate. Subtype was also adjusted as covariate for the subset of samples consisted of multiple subtypes. ALL: all subtypes. Non-TN: all subtypes except for TN. This data was used in Figures 4A-D.

Supplementary Table 3: Sample statistics for TIL changes over time

Time	TIL change	pCR cohort	RD cohort	p-value	Subtype
T1-T2	Increase	26 (72%)	32 (52%)	0.05626	ALL
	Decrease	10 (28%)	30 (48%)		
T1-T3	Increase		9 (35%)	NA	ALL
	Decrease		17 (65%)		
T2-T3	Increase		9 (39%)	NA	ALL
	Decrease		14 (61%)		
T1-T2	Increase	12 (80%)	15 (83%)	1	TN
	Decrease	3 (20%)	3 (17%)		
T1-T3	Increase		6 (50%)	NA	TN
	Decrease		6 (50%)		
T2-T3	Increase		5 (46%)	NA	TN
	Decrease		6 (54%)		
T1-T2	Increase	14 (67%)	17 (38%)	0.0618	Non-TN
	Decrease	7 (33%)	27 (62%)		
T1-T3	Increase		3 (21%)	NA	Non-TN
	Decrease		11 (79%)		
T2-T3	Increase		4 (33%)	NA	Non-TN
	Decrease		8 (67%)		
T1-T2	Increase	7 (70%)	2 (20%)	0.06978	HER2+/ER-
	Decrease	3 (30%)	8 (80%)		
T1-T3	Increase		1 (17%)	NA	HER2+/ER-
	Decrease		5 (83%)		
T2-T3	Increase		2 (50%)	NA	HER2+/ER-
	Decrease		2 (50%)		
T1-T2	Increase	5 (55%)	7 (58%)	1	ER+/HER2+
	Decrease	4 (45%)	5 (42%)		
T1-T3	Increase			NA	ER+/HER2+
	Decrease				
T2-T3	Increase			NA	ER+/HER2+
	Decrease				
T1-T2	Increase	2 (100%)	8 (36%)	0.163	ER+/HER2-
	Decrease	0 (0%)	14 (64%)		
T1-T3	Increase		2 (25%)	NA	ER+/HER2-
	Decrease		6 (75%)		
T2-T3	Increase		2 (25%)	NA	ER+/HER2-
	Decrease		6 (75%)		

A table showing the numbers and percentages of tumor samples exhibiting either increases or decreases in TIL density between two treatment times in different subsets of patients grouped by subtype (row) and treatment outcome (column). P-

value: statistical significance of the difference between two treatment outcomes as determined by two-sided Fisher's exact test.

Supplementary Table 4: Comparison statistics for immune cell fraction estimates

Cell type	Fold change (t-statistic)	p-value	FDR (q-value)	Comparison
B.cells.naive	-0.137418061	0.889934724	0.889934724	T1 vs T2
B.cells.memory	0.159531978	0.872366825	0.889934724	T1 vs T2
T.cells.CD8	2.737364425	0.007208332	0.015858331	T1 vs T2
T.cells.follicular.helper	-4.780478983	7.33E-06	4.03E-05	T1 vs T2
Macrophages.M0	-2.124194078	0.035205052	0.064542596	T1 vs T2
Macrophages.M1	-2.886374326	0.00471935	0.012978211	T1 vs T2
Macrophages.M2	-0.15052432	0.879515515	0.889934724	T1 vs T2
T.Cells.CD4.Memory	5.003047238	3.18E-06	3.50E-05	T1 vs T2
NK.cells	1.064943543	0.285422651	0.448521309	T1 vs T2
Mast.cells	-0.166722195	0.866668103	0.889934724	T1 vs T2
Dendritic.cells	2.964379635	0.003759568	0.012978211	T1 vs T2
B.cells.naive	-2.189027691	0.03368877	0.190776218	T2 vs T3
B.cells.memory	-1.889352943	0.06329834	0.232093915	T2 vs T3
T.cells.CD8	-0.564217551	0.553895017	0.761605648	T2 vs T3
T.cells.follicular.helper	1.703947117	0.091475653	0.251558047	T2 vs T3
Macrophages.M0	0.108848412	0.908900861	0.908900861	T2 vs T3
Macrophages.M1	-1.234627229	0.214252054	0.363608881	T2 vs T3
Macrophages.M2	1.148151399	0.23138747	0.363608881	T2 vs T3
T.Cells.CD4.Memory	-2.105398057	0.034686585	0.190776218	T2 vs T3
NK.cells	0.158036318	0.868063572	0.908900861	T2 vs T3
Mast.cells	1.548131165	0.123148079	0.270925774	T2 vs T3
Dendritic.cells	0.295298139	0.76245294	0.908900861	T2 vs T3

A table showing the statistics related to the changes in different immune cell fractions between two treatment times, T1 vs. T2 or T2 vs. T3. Statistical significance was calculated using LMER adjusting for tumor purity and subtype as covariates. This data was used in Supplementary Figures 5b-c.

Supplementary Table 5: Association statistics of immune states

Feature	Feature Type	Hot (H)	Warm (W)	Cold (C)	p-value	Cohort
T1	treatment time	44 (39%)	41 (36%)	29 (25%)	9.37E-06	ALL
T2	treatment time	51 (57%)	24 (27%)	15(16%)		ALL
T3	treatment time	1 (3%)	12 (43%)	15 (54%)		ALL
RD	treatment response	51 (39%)	43 (33%)	38 (29%)	8.46E-04	ALL
pCR	treatment response	44 (61%)	22 (31%)	6 (8%)		ALL
ER+/HER2-	subtype	12 (24%)	14 (27%)	25 (49%)	0.001671	ALL
ER+/HER2+	subtype	18 (43%)	14 (33%)	10 (24%)		ALL
HER2+/ER-	subtype	19 (44%)	15 (35%)	9 (21%)		ALL
TN	subtype	47 (49%)	34 (35%)	15 (16%)		ALL
RD	treatment response	23 (49%)	11 (23%)	13 (28%)	3.77E-03	T2
pCR	treatment response	20 (83%)	4 (17%)	0 (0%)		T2
ER+/HER2-	subtype	4 (25%)	4 (25%)	8 (50%)	1.76E-05	T2
ER+/HER2+	subtype	8 (50%)	7 (44%)	1 (6%)		T2
HER2+/ER-	subtype	7 (58%)	3 (25%)	2 (17%)		T2
TN	subtype	21 (95%)	1 (5%)	0 (0%)		T2

Estimates of the statistical association between key features and immune states in either the entire cohort (All) or on-treatment samples (T2). Statistical significance was determined using two-sided Chi-squared test. This data was used in Supplementary Figures 6c-e.

Supplementary Table 6: Comparison statistics for on-treatment immune state transitions

Feature	C-C	C-H	C-W	H-C	H-H	H-W	W-H	W-W	p-value
ER+/HER2-	7 (44%)	0 (0%)	2 (13%)	1 (6%)	3 (18%)	0 (0%)	1 (6%)	2 (13%)	0.000798
ER+/HER2+	1 (6%)	2 (13%)	3 (19%)	0 (0%)	5 (31%)	2 (12%)	1 (6%)	2 (13%)	
HER2+/ER-	2 (15%)	1 (8%)	0 (0%)	0 (0%)	4 (31%)	2 (15%)	3 (23%)	1 (8%)	
TN	0 (0%)	1 (4%)	1 (4%)	0 (0%)	7 (29%)	0 (0%)	15 (63%)	0 (0%)	
pCR	0 (0%)	2 (8%)	1 (4%)	0 (0%)	9 (38%)	2 (8%)	9 (38%)	1 (4%)	0.09658
RD	10 (22%)	2 (4.5%)	5 (11%)	1 (2%)	10 (22%)	2 (4.5%)	11 (25%)	4 (9%)	

A table showing the numbers and percentages of samples that exhibited different statuses of immune state transitions during treatment (T1 vs. T2) in different subsets of samples grouped by subtypes and treatment outcomes. P-value: statistical significance of the differences between subtypes or treatment outcomes as determined by two-sided Chi-squared test.
