# Supplementary material for: Chemotherapy induces dynamic immune responses in breast cancers that impact treatment outcome
Source: Nat Commun. 2020 Dec 2;11:6175. doi: 10.1038/s41467-020-19933-0 (PMC7710739; doi:10.1038/s41467-020-19933-0)
Supplement: Supplementary file 2 — Descriptions of Additional Supplementary Files [file 41467_2020_19933_MOESM2_ESM.docx]

**Descriptions of Additional Supplementary Files**

**Supplementary Data 1: Sample annotation**

Sample annotation for all the tumor samples in our cohort. Columns describe patient and sample identifiers (A, B), treatment times (C), breast cancer subtypes (D), gene expressions as log2TPM for key marker genes – *ESR1*, *ERBB2*, *PGR* (G-I), tumor purity (J), Ki67 status (K), TNM stage (L), neoadjuvant chemotherapy (NAC) regimen (M), NAC treatment outcome (N) and sample pairing status (O).

**Supplementary Data 2: Geneset enrichment analysis of DE genes**

Geneset enrichment analysis results based on hypergeometric tests for three clusters of differentially expressed genes – C1, C2 and C3.

**Supplementary Data 3: DE gene signatures mapped to DE gene clusters**

Significant DE signatures identified through comparison analysis of GSVA scores vs. treatment times. Also shown are mappings to genesets significantly enriched in DE gene clusters (H-J). Statistical significance was determined using LMER adjusting for tumor purity and subtype as covariates. Columns D-G show the sign*-log10(p-value) based on the sign of the t-statistics.

**Supplementary Data 4: TIL density and immune features**

TIL density, strom TIL scores and immune state classifications for all tumor samples.

**Supplementary Data 5: Geneset enrichment in NMF factors**

Genesets significantly enriched in genes associated with NMF factors F1-F14 based on hypergeometric test.

**Supplementary Data 6: Independent associations of immune features vs. clinical factors**

Estimates of independent associations between clinical factors and immune features of T1 or T2 samples. Columns C-I represent –log10(p-value) of the association between each feature (row) and clinical factor (column) after adjusting for the confounding effect of other factors. This data was used in Figure 7b.

**Supplementary Data 7: Elastic net analysis for predicting treatment outcome**

A table showing the elastic net and bootstrapping analysis results. Columns describe the immune feature name and type, the regression coefficient, and variable usage determined by the percentage of runs in which the elastic net model selected the variable to predict treatment outcome. This data was used in Figures 7b-c.
